# Electrophysiological abnormalities in induced pluripotent stem cell‐derived cardiomyocytes generated from Duchenne muscular dystrophy patients

**DOI:** 10.1111/jcmm.14124

**Published:** 2019-01-08

**Authors:** Binyamin Eisen, Ronen Ben Jehuda, Ashley J. Cuttitta, Lucy N. Mekies, Yuval Shemer, Polina Baskin, Irina Reiter, Lubna Willi, Dov Freimark, Mihaela Gherghiceanu, Lorenzo Monserrat, Michaela Scherr, Denise Hilfiker‐Kleiner, Michael Arad, Daniel E. Michele, Ofer Binah

**Affiliations:** ^1^ Department of Physiology, Biophysics and Systems Biology, Rappaport Faculty of Medicine Technion – Israel Institute of Technology Haifa Israel; ^2^ Department of Biotechnology Technion – Israel Institute of Technology Haifa Israel; ^3^ Department of Molecular and Integrative Physiology University of Michigan Ann Arbor Michigan; ^4^ Leviev Heart Center, Sheba Medical Center Ramat Gan Israel; ^5^ Sackler Faculty of Medicine Tel Aviv University Tel Aviv Israel; ^6^ Victor Babes National Institute of Pathology Bucharest Romania; ^7^ Health in Code Corunna Spain; ^8^ Department of Hematology, Hemostasis, Oncology and Stem Cell Transplantation Hannover Medical School Hannover Germany; ^9^ Department of Cardiology and Angiology, Hannover Medical School Hannover Germany

**Keywords:** arrhythmia, dilated cardiomyopathy, Duchenne muscular dystrophy, induced pluripotent stem cell‐derived cardiomyocytes, X chromosome inactivation

## Abstract

Duchenne muscular dystrophy (DMD) is an X‐linked progressive muscle degenerative disease, caused by mutations in the *dystrophin* gene and resulting in death because of respiratory or cardiac failure. To investigate the cardiac cellular manifestation of DMD, we generated induced pluripotent stem cells (iPSCs) and iPSC‐derived cardiomyocytes (iPSC‐CMs) from two DMD patients: a male and female manifesting heterozygous carrier. Dystrophin mRNA and protein expression were analysed by qRT‐PCR, RNAseq, Western blot and immunofluorescence staining. For comprehensive electrophysiological analysis, current and voltage clamp were used to record transmembrane action potentials and ion currents, respectively. Microelectrode array was used to record extracellular electrograms. X‐inactive specific transcript (XIST) and dystrophin expression analyses revealed that female iPSCs underwent X chromosome reactivation (XCR) or erosion of X chromosome inactivation, which was maintained in female iPSC‐CMs displaying mixed X chromosome expression of wild type (WT) and mutated alleles. Both DMD female and male iPSC‐CMs presented low spontaneous firing rate, arrhythmias and prolonged action potential duration. DMD female iPSC‐CMs displayed increased beat rate variability (BRV). DMD male iPSC‐CMs manifested decreased *I*_f_ density, and DMD female and male iPSC‐CMs showed increased *I*_Ca,L _density. Our findings demonstrate cellular mechanisms underlying electrophysiological abnormalities and cardiac arrhythmias in DMD.

## 1 INTRODUCTION

DMD caused by mutations in the *dystrophin* gene, is an X‐linked muscle degenerative disease present in 1.7‐2.1 of 10 000 male births.^1^, ^2^, ^3^ Homozygous symptomatic females are rare and heterozygous females are mostly unaffected carriers, yet ~20% are symptomatic manifesting carriers with later and milder disease progression than male patients.^4^, ^5^, ^6^ In DMD patients dystrophin mutations lead to destabilization of the dystrophin glycoprotein complex (DGC) and sarcolemma instability resulting in functional deficits.^7^ Dystrophin also plays a role in anchoring dystrophin‐associated proteins to the sarcolemma, including nitric oxide synthase (nNOS).^8^ Absence of functional dystrophin ultimately leads to degeneration and death of skeletal and cardiac muscle, being replaced by fibrous tissue.^9^ DMD symptoms begin at early childhood, mainly including muscle fatigue. With age, the disease progresses and wheelchair assistance is needed in early teen years. Eventually, respiratory function is compromised and requiring mechanical support. Cardiac dysfunction usually manifests during late teenage or early twenties, but ultimately dilated cardiomyopathy (DCM) and congestive heart failure develop with ventricular and supraventricular arrhythmias. Finally, patients progress to death because of respiratory or cardiac failure.^10^

In female embryos, one of the X‐chromosomes is randomly inactivated leading to mosaicism of maternal and paternal X‐linked alleles explaining why heterozygous females are usually less affected or asymptomatic. iPSCs enable generation of human disease models to study underlying pathomechanisms in a cell type specific level. Previous studies investigating the effect of reprogramming on X chromosome status suggest three possible outcomes: (1) X chromosome inactivation (XCI) status is maintained throughout the reprogramming process; (2) inactivated X chromosome is reactivated; (3) inactivated X chromosome is eroded.^11^, ^12^, ^13^, ^14^, ^15^, ^16^, ^17^, ^18^

To investigate the electrophysiological abnormalities caused by dystrophin mutations in cardiomyocytes, we generated iPSCs from two DMD patients: a male and a female heterozygous manifesting carrier. X inactivation status and dystrophin gene expression were analysed in iPSCs and iPSC‐CMs generated from healthy controls and patients of both sexes with regard to genetic, molecular and functional abnormalities. In support of the hypothesis that dystrophin‐mutated iPSC‐CMs from the mutant male and the heterozygous female carrier exhibit key features of DMD, mutated cardiomyocytes displayed molecular, genetic and electrophysiological abnormalities, including arrhythmias.

## 2 METHODS

See Supporting Information for detailed Methods.

Dermal biopsies were obtained from a 50‐year‐old DMD female manifesting patient carrying a deletion of exons 8‐12 (ex.8_12del) and a 32‐year‐old DMD male patient carrying a substitution of cytosine to thymine (c.5899C>T) constituting a premature stop codon. The donors signed a consent form according to approval #7603‐09‐SMC by the Helsinki Committee for Experiments on Human Subjects at Sheba Medical Center, Ramat Gan, Israel. See Supporting Information for Methods including, patients' clinical history, iPSCs generation and characterization,^19^ karyotype analysis, teratoma formation, genotyping and pluripotency evaluation by immunofluorescence staining and flow cytometry. Differentiation into cardiomyocytes was performed according to the directed differentiation by modulating Wnt/β‐catenin signalling as previously described.^20^ Transmission electron microscopy was used to evaluate ultrastructural features of iPSC‐CMs. See Supporting Information for protein and RNA expression analyses.

### 2.1 Electrophysiological experiments

Action potentials were recorded from small cardiomyocyte clusters and single cells in whole‐cell configuration. The pacemaker current *I*_f_ was recorded from single cardiomyocytes (enzymatically dissociated) in the presence of 500 μM BaCl_2_ to block *I*_K1_. To record *I*_f_, the membrane was clamped at 15 seconds intervals, from a holding potential of −40 mV to −120 mV in 10 mV steps for 2 seconds pulse durations.^21^ To record L‐Type Ca^2+^ current (*I*_Ca,L_), the membrane was clamped at 200 ms depolarizing steps, from a holding potential of −70 mV ranging from −40 to +40 mV after a 20 ms −40 mV pulse for inactivation of Na^+^ currents.^22^ Axopatch 200B, Digidata 1322 or 1440 and pClamp10 (Molecular Devices, Sunnyvale, CA, USA) were used for data amplification, acquisition and analysis. For current recording, pipette capacitance compensation was adjusted, whole cell capacitance was measured and series resistance compensation was adjusted to 80%; finally, current density was calculated. Extracellular electrograms were recorded for 1000‐1800 seconds from spontaneously contracting iPSC‐CMs clusters by using the Micro Electrode Array (MEA) apparatus (Multi Channels Systems, Reutlingen, Germany).

### 2.2 Statistical analysis

Results are presented as mean ± SEM. Comparisons between Male‐DMD, Female‐DMD and control iPSC‐CMs were performed with one‐way or two‐way ANOVA followed by Holm‐Sidak test using SigmaPlot 12.0 software (Systat Software International, San Jose, CA, USA). A value of *P* < 0.05 was considered statistically significant.

## 3 RESULTS

### 3.1 Clinical characterization of the DMD male patient and heterozygous symptomatic female patient

The male DMD patient suffered from muscular dystrophy from early childhood and was diagnosed with DCM at the age of 17. At age 25, he was hospitalized with respiratory insufficiency primarily because of combined respiratory and heart failure, and required tracheostomy and prolonged ventilation. At that time, his LVEF was 15% and the ECG showed sinus rhythm with narrow QRS and QE pattern in L1, AVL and V2‐3. At age 30, he was respirator‐dependent but his heart failure was reasonably controlled. The echo‐Doppler showed LVEDD 58 mm, LVEF 20%, moderate mitral insufficiency and normal right ventricular function. At age 33, a routine Holter‐ECG showed for the first time episodes of non‐sustained ventricular tachycardia of 3‐12 beat at a rate of up to 200/minute. Two years later, because of repeated episodes of ventricular flutter deteriorating into ventricular fibrillation, in order to save his life an ICD was implanted.

The female patient, a manifesting carrier, presented at age 42 with proximal muscle weakness and creatine kinase elevation. Her son diagnosed with DMD expired at age 16. A year later, her echo‐Doppler showed a biventricular dysfunction with left ventricular dimension of 65 mm, left ventricular ejection fraction of 30% and moderate to severe mitral insufficiency. Her ECG showed low voltage with left axis deviation and narrow QRS, she suffered from paroxysms of atrial fibrillation with rapid ventricular response and non‐sustained ventricular tachycardia. An ICD was implanted, but despite heart failure therapy, her condition continued to deteriorate. Cardio‐respiratory exercise test showed VO_2_max of 6 ml/kg/min (33% of normal value) resulting from primarily cardiovascular limitation, but the patient declined heart transplantation. At age 49, she was in NYHA IV. AV nodal ablation and upgrading to CRTD was required because of atrial fibrillation with rapid ventricular response. Echo‐Doppler showed severe biventricular dysfunction with LVEF of 20% and severe tricuspid regurgitation. The muscle weakness progressed to complete inability to walk. The patient expired at age 51 because of end‐stage heart failure associated with renal insufficiency.

### 3.2 Molecular characterization of DMD iPSCs and iPSC‐CMs

iPSCs were generated from dermal fibroblast. For iPSCs characterization, FACS and immunostaining validated pluripotent markers expression (Figures S1 and S2). Teratoma assay demonstrated iPSCs ability to differentiate into the three germ layers (Figure S1). Karyotype was preserved throughout the reprogramming process, and Sanger sequencing confirmed the mutations in the patients iPSCs (Figure S1). For control, iPSCs and iPSC‐CMs were generated from four different healthy female and male donors with varying genetic backgrounds, thus markedly strengthening the comparisons between the mutated and control populations.

**Figure 1:**
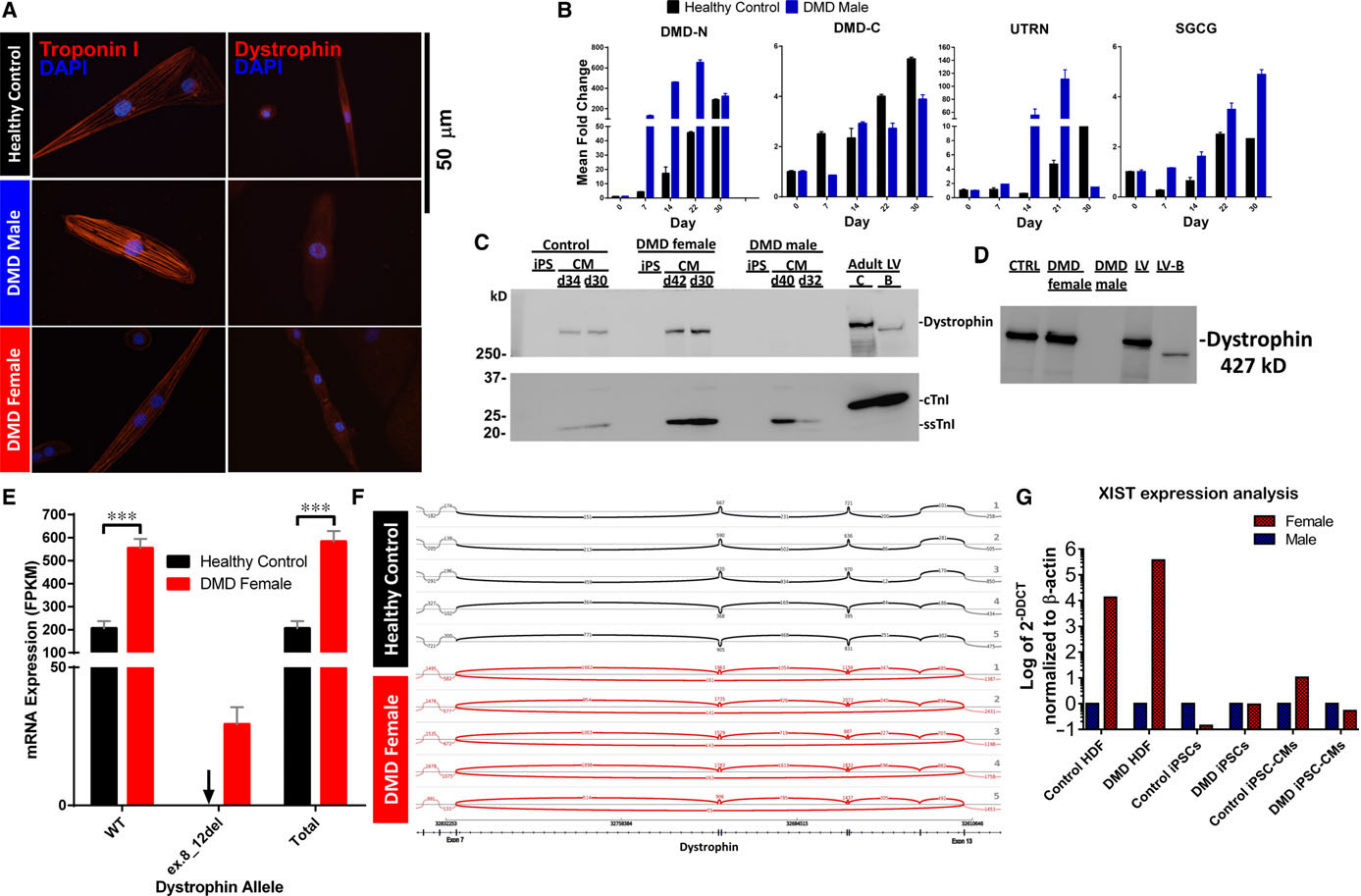
Induction of muscle specific dystrophin and dystrophin‐glycoprotein complex (DGC) expression during iPSC‐CM differentiation. (A) Immunofluorescence staining of iPSC‐CMs >30 d shows presence of sarcomere localized Troponin I and dystrophin expression (C‐terminal antibody). Dystrophin is reduced in all DMD iPSC‐CMs and some DMD female iPSC‐CMs. (B) mRNA expression of muscle specific 427 kD dystrophin (DMD‐N) and DGC components in DMD male and control iPSC‐CMS; n = 2‐5 independent differentiations for each time point. (C) Western blotting of full‐length dystrophin, slow skeletal troponin I (ssTnI, 22 kD), and cardiac troponin I (cTnI, 26 kD) in control and DMD iPSC‐CMs, and adult left ventricle (LV) tissue lysates. iPSC‐CMs were collected at >30 d (d). Mean dystrophin/TnI ratio by densitometry was 6.3, 1.1 and <0.02 in control, DMD female and male preparations, respectively. LV adult tissue lysates were generated from adult control (C) and Becker muscular dystrophy (BMD) patients with an in‐frame dystrophin deletion leading to truncated dystrophin (B). (D) Wheat germ agglutinin (WGA) purification enriches the glycosylated proteins of the dystrophin‐glycoprotein complex and dystrophin in control (CTRL) iPSC‐CMs, DMD female iPSC‐CMs but is absent in DMD male iPSC‐CM. LV tissue samples from adult control (LV) and BMD (LV‐B) patients show enrichment of dystrophin as expected. Mixed expression of dystrophin (WT and mutated (ex.8_12del) alleles in the DMD female iPSC‐CM population**.** (E) NGS analysis of the dystrophin alleles mRNA expression in DMD female and control iPSC‐CMs. Fragments per kilobase of exon per million reads mapped (FPKM) values were normalized by library size and corrected by enrichment factor. Black arrow indicates zero expression of the mutated allele in control iPSC‐CMs samples. Control n = 5; DMD female, n = 5. Two‐way ANOVA followed by Holm‐Sidak post‐hoc analysis. ***P < 0.001. (F) Sashimi plot of the WT allele RNA in control iPSC‐CMs (top) and both WT and mutated alleles RNA in DMD female iPSC‐CMs (bottom). (G) Expression of XIST RNA in female compared to male human fibroblasts (HF), iPSC and iPSC‐CM as determined by qRT‐PCR. Expression of XIST was normalized to ß‐actin and relative expression levels are shown as 2^−△△CT^ compared to the respective male control

iPSCs differentiation resulted in cardiomyocytes and myofibrillogenesis (Figure 1A) depicted by troponin I expression (at 30 days) and spontaneous contractions. Ultrastructural analyses did not reveal any differences between the experimental groups, including changes in sarcomere length (Figure S4). Using dystrophin exon‐specific qRT‐PCR, control and DMD male iPSC‐CMs produced robust induction of full‐length 427 kD muscle isoform of dystrophin mRNA, while total dystrophin transcript measured by a common C‐terminal exon only increased modestly overtime post‐differentiation. Utrophin mRNA expression was also induced during differentiation but was suppressed during late iPSC‐CMs development. Muscle‐specific γ‐sarcoglycan was also expressed following differentiation in control and DMD male iPSC‐CMs. By Western blotting (WB), full‐length 427 kD dystrophin was detected in control and DMD female iPSC‐CMs, but undetectable in DMD male iPSC‐CMs, confirming a protein null allele in this patient (Figure 1C). All clones manifested slow skeletal TnI (ssTnI), the neonatal isoform of troponin I in iPSC‐CMs indicating induction of sarcomerogenesis. To test assembly of dystrophin with the DGC, membrane preparations were prepared from iPSC‐CMs, solubilized and passed over wheat germ agglutinin affinity columns, which demonstrated that dystrophin co‐purified with glycoprotein fractions similar to adult heart tissue from normal and Becker muscular dystrophy patients with a truncated dystrophin allele (Figure 1D).

### 3.3 Genetic characterization of the female manifesting carrier

Previous studies suggested an inactive X chromosome may undergo reactivation (XCR) or erosion during human iPSCs (hiPSCs) derivation^23^, ^24^, ^25^ while others proposed that XCI is maintained throughout the reprograming process.^26^ Given that hearts of females carrying dystrophin mutations in humans and mice show random XCI and mosaic cellular dystrophin expression,^27^ we aimed to evaluate the X chromosome status during reprogramming and differentiation. XIST is a long non‐coding RNA on the X chromosome that acts as a major effector of the X inactivation process.^17^, ^28^ Expression analysis demonstrated the absence of XIST RNA in iPSCs, indicating that XCR or erosion of XCI occurred during reprogramming of DMD and control female dermal fibroblasts. In agreement with previous reports,^24^, ^25^, ^29^ XIST expression patterns were maintained during differentiation of iPSCs into iPSC‐CMs (Figure 1G). As XIST does not fully correlate with XCI in iPSCs,^17^, ^30^ thus preventing distinction between XCR and erosion of XCI, X chromosome status could not be determined definitively. To investigate the expression pattern of dystrophin in iPSC‐CMs, we used next generation sequencing (NGS) which demonstrated (Figure 1E,F) mixed dystrophin expression in the female iPSC‐CM population, displaying both mutated and WT dystrophin alleles. Specifically, dystrophin transcripts expressed as mRNA fragments per kilobase of exon per million reads mapped (FPKM) mean values were 29.35 and 554.04 for the mutated and WT alleles, respectively, indicating mixed expression of both alleles in the DMD female iPSC‐CMs. In contrast, as expected, control iPSC‐CMs express the WT allele only, with a FPKM mean value of 205.49 (Figure 1E).

### 3.4 Electrophysiological abnormalities in female and male DMD iPSC‐CMs

**Figure 2:**
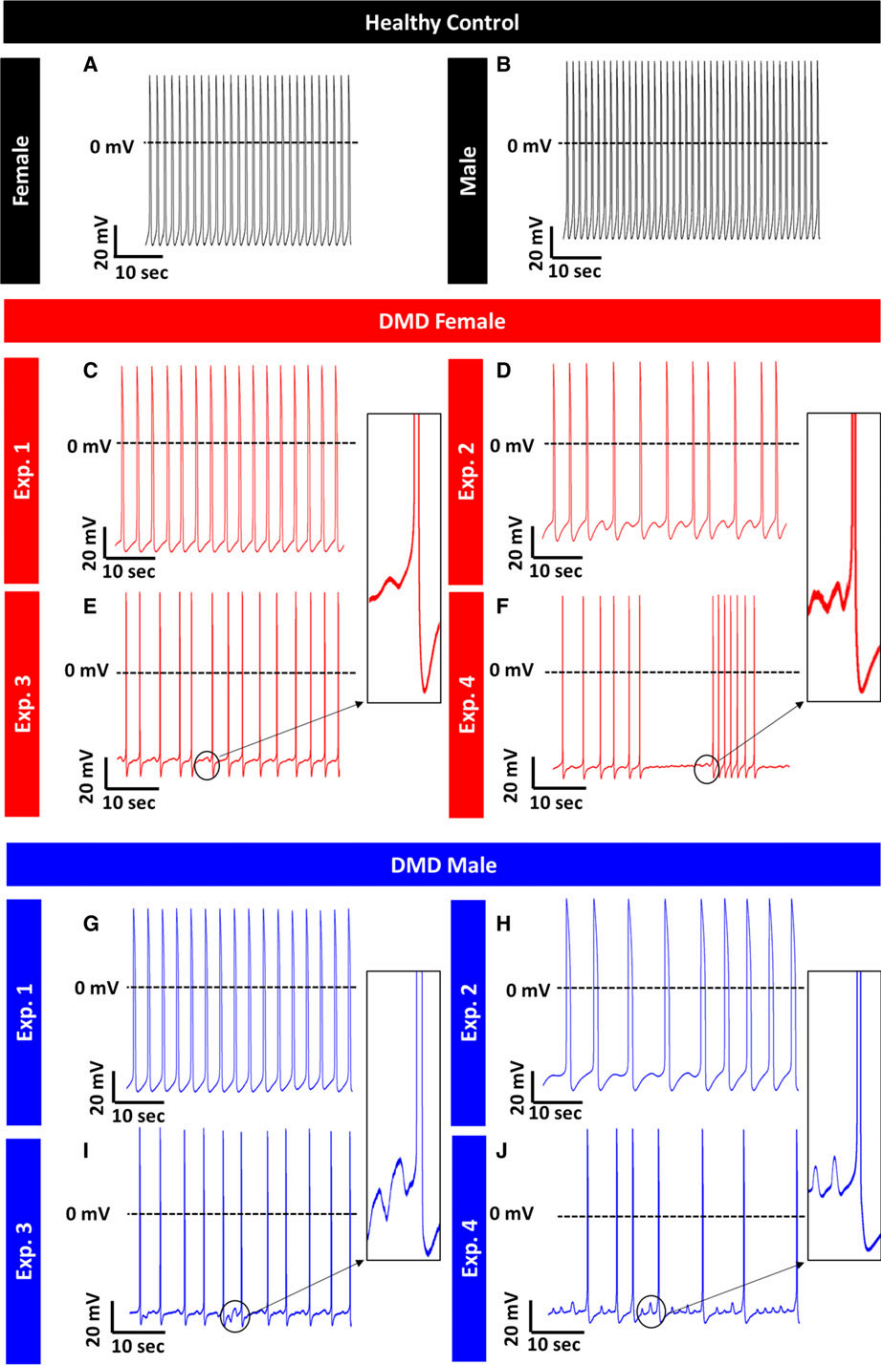
Representative spontaneous action potential recordings from control and DMD iPSC‐CMs. (A, B) Control iPSC‐CMs do not manifest arrhythmias during spontaneous activity. (C‐F) DMD female iPSC‐CMs display arrhythmogenic firing pattern including delayed afterdepolarizations (DADs) and oscillatory prepotentials in 52% of spontaneous recordings (panels D‐F); (G‐J) DMD male iPSC‐CMs display arrhythmogenic firing pattern including DADs and OPPs in 17% of spontaneous recordings (panels H‐J). Control n = 25; DMD female, n = 21; DMD male, n = 23

**Figure 3:**
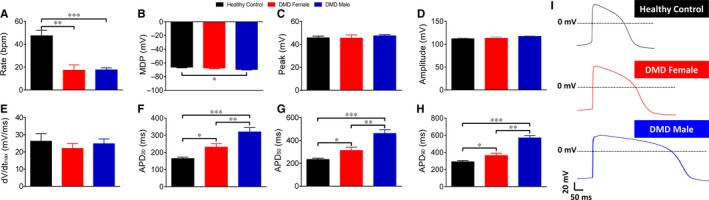
Action potential parameters of spontaneously firing DMD and control iPSC‐CMs. (A‐H) Action potential parameters of iPSC‐CMs. (A) Beat rate; (B) Maximal diastolic potential (MDP); (C) Action potential peak; (D) Action potential amplitude; (E) Maximal rate of Phase 0 depolarization (dV/dt_max_); (F‐H) Action potential duration at 20/50/90% of repolarization (APD_20/50/90_). (I) Representative action potentials displaying prolonged APDs in DMD iPSC‐CMs compared to control iPSC‐CMs. Control n = 25; DMD female, n = 21; DMD male, n = 23. One‐way ANOVA followed by Holm‐Sidak post‐hoc analysis. *P < 0.05, **P < 0.01, ***P < 0.001

**Figure 4:**
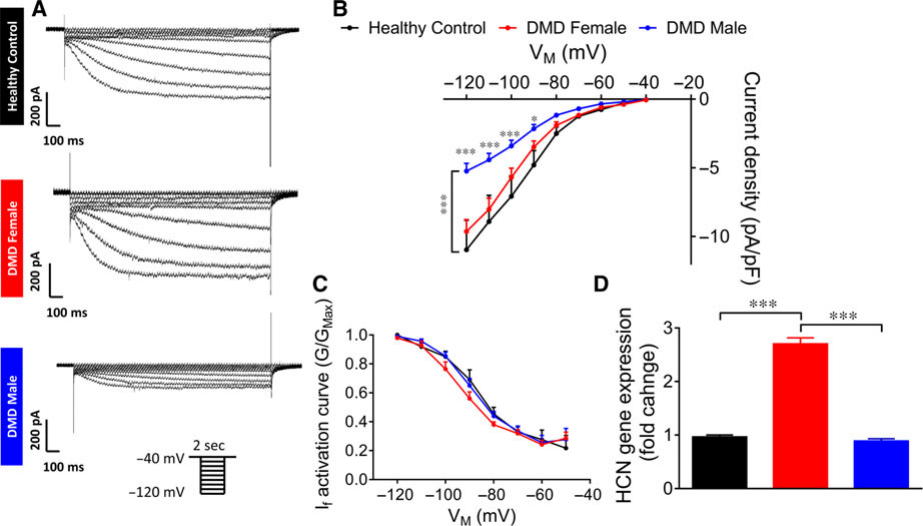
*I*_f_ in control and DMD iPSC‐CMs. (A) Representative recordings of *I*_f_ in control and DMD iPSC‐CMs. (B) *I*_f_ Current‐Voltage (I‐V) curve in control and DMD iPSC‐CMs. (C) *I*_f_ activation curve. Control n = 8; DMD female, n = 10; DMD male, n = 12. Two‐way ANOVA followed by Holm‐Sidak post‐hoc analysis. (D) Gene expression of HCN (isoforms HCN2 and HCN4) in control and DMD iPSC‐CMs. Duplicates/triplicates of independent differentiations were used for each clone: control, DMD female and DMD male; n = 3 for each experimental condition. One‐way ANOVA followed by Holm‐Sidak post‐hoc analysis. *P < 0.05, ***P < 0.001

Common pathophysiological phenomena in DMD patients are supraventricular and ventricular arrhythmias, the latter being the second most common cause of death in these patients.^31^, ^32^ Accordingly, we investigated action potential properties and specific ionic currents in control and DMD iPSC‐CMs generated from multiple independent differentiations. A prominent electrophysiological abnormality in DMD iPSC‐CMS was slower spontaneous firing rates compared to control cardiomyocytes; female—21.6 bpm, male—17.6 bpm, control—47.4 bpm (Figures 2, 3A). To investigate the mechanism underlying this finding, we compared the properties of *I*_f_ (a key pacemaker current) in control versus DMD iPSC‐CMs. As illustrated by representative current traces (Figure 4A) and I‐V relations (Figure 4B), while DMD female iPSC‐CMs did not differ from control, *I*_f_ density in DMD male iPSC‐CMs was smaller (*P* < 0.001). No differences were observed in current activation relations of the three groups (Figure 4C), implying similar channel properties. To decipher the mechanisms underlying the differences in *I*_f_ I‐V relations among the groups, we measured the expression of the hyperpolarization‐activated cyclic nucleotide‐gated channel—isoforms 2 and 4 (HCN2 and HCN4) by means of qRT‐PCR analysis. These experiments demonstrated increased expression of HCN2 and HCN4 in DMD female iPSC‐CMs compared to control and DMD male iPSC‐CMs (Figure 4D).

#### 3.4.1 Arrhythmias in DMD iPSC‐CMs

In addition to the slow firing rate, DMD female and male iPSC‐CMs displayed voltage oscillations, which resemble delayed afterdepolarizations (DADs) and oscillatory prepotentials (OPPs) during action potential recordings. As demonstrated in Figure 2, DMD female and male iPSC‐CMs presented oscillatory depolarization in 52% and 17% of spontaneous recordings, respectively (Figure 2), while control iPSC‐CMs had no arrhythmias.

#### 3.4.2 Action potential parameters

As seen in Figure 3, action potential parameters of DMD female and male iPSC‐CMs differed from control. Mean action potential durations at 20/50/90% repolarization (APD_20_/APD_50_/APD_90_) were longer than control; further, the mean APD_20_/APD_50_/APD_90_ were longer in DMD male than in female iPSC‐CMs (Figure 3F‐H). Importantly, markedly prolonged APD_90_ (>400 ms) was observed in 29% and 100% of DMD female and male iPSC‐CMs, respectively. Finally, mean maximal diastolic potential was more negative in DMD male iPSC‐CMs (−69.6 mV) than control (−66.2 mV) (Figure 3B).

**Figure 5:**
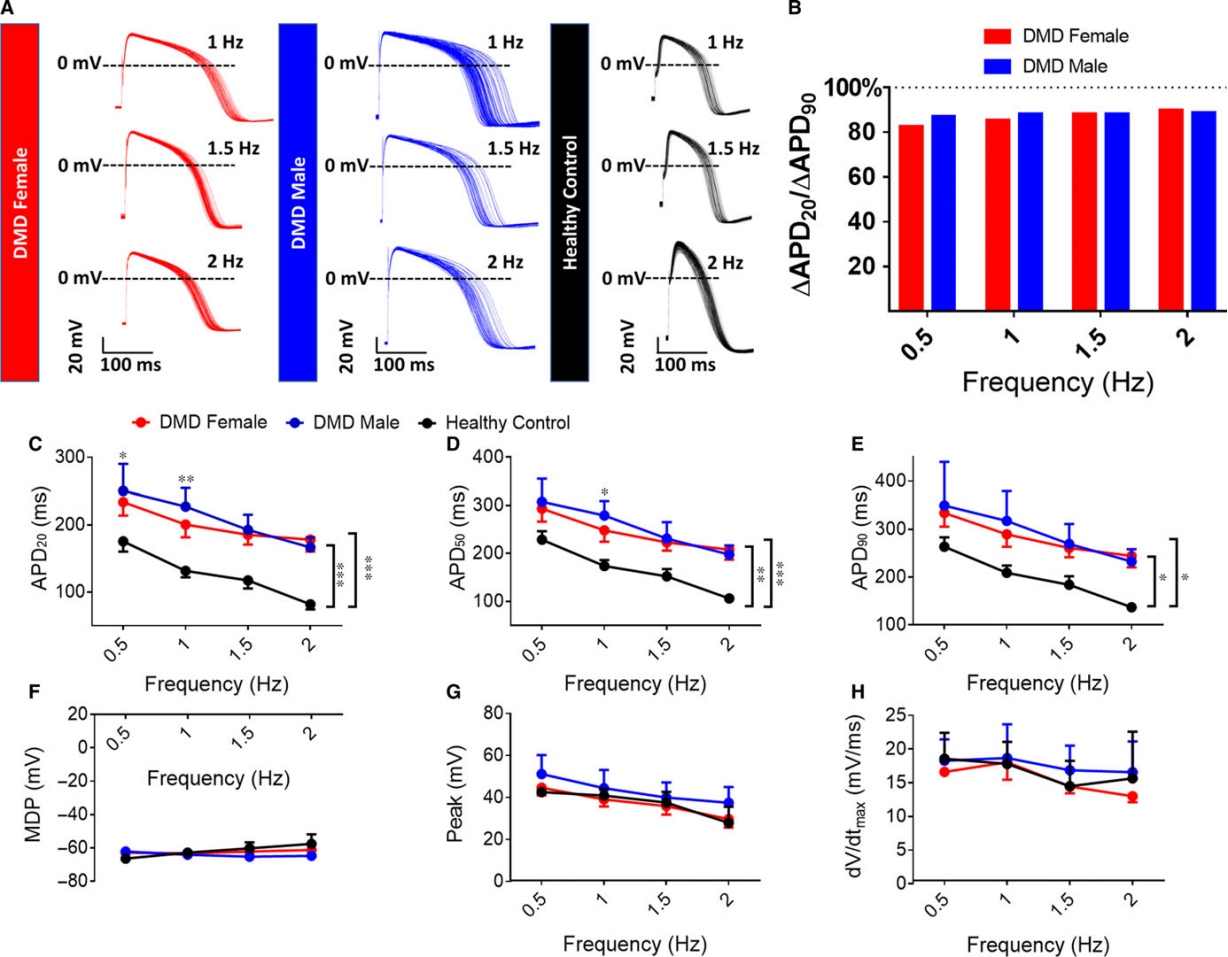
Action potential parameters of paced control and DMD iPSC‐CMs. (A) Representative paced action potentials displaying prolonged APDs in DMD iPSC‐CMs compared to control. (B) The ratio between the difference in APD20 and the difference in APD90 of DMD and control iPSC‐CMs demonstrating the main prolongation during early repolarization. Control n = 8; DMD female, n = 8; DMD male, n = 9. Action potential amplitude. (C‐H) Action potential parameters of iPSC‐CMs under different pacing frequencies. (C‐E) Action potential duration at 20/50/90% of repolarization (APD20/50/90); (F) Maximal diastolic potential (MDP); (G) Action potential peak; (H) Maximal rate of phase 0 depolarization (dV/dtmax). Two‐way ANOVA followed 5 by Holm‐Sidak post‐hoc analysis. *P < 0.05, **P < 0.01, ***P < 0.001

To neutralize the effect of slow firing rate in DMD cardiomyocytes on APD, we measured action potential parameters in paced single cell cardiomyocytes. In agreement with the results from spontaneously firing cardiomyocytes, APD_20_/APD_50_/APD_90_ were prolonged in DMD female and male iPSC‐CMs (Figure 5A, 5‐E), while other parameters were comparable to control (Figure 5B). To identify the specific action potential phase responsible for APD prolongation, we calculated the ratio between APD at different stages of repolarization and APD_90_; the APD contributing mostly to prolongation was APD_20_, implying involvement of early repolarization phase (ie APD_20_; Figure 5B).

#### 3.4.3 Increased *I*_Ca,L_ density in DMD iPSC‐CMs

**Figure 6:**
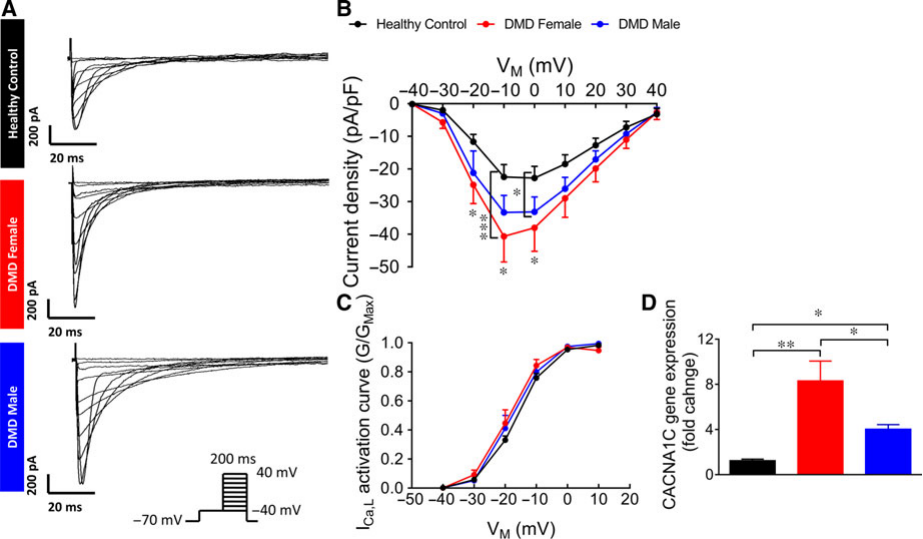
*I*_Ca,L_ in control and DMD iPSC‐CMs. (A) Representative *I*_Ca,L_ recordings in control and DMD iPSC‐CMs. (B) *I*_Ca,L_ Current‐Voltage (I‐V) curve in control and DMD iPSC‐CMs. (C) *I*_Ca,L _activation curve. Control n = 9; DMD female, n = 10; DMD male, n = 6. Two‐way ANOVA followed by Holm‐Sidak *post‐hoc* analysis. (D) Gene expression of CACNA1C in control and DMD iPSC‐CMs. Duplicates/triplicates of independent differentiations were used for each clone: control, DMD female and DMD male; n = 3 for each experimental condition. One‐way ANOVA followed by Holm‐Sidak post‐hoc analysis. *P < 0.05, **P < 0.01, ***P < 0.001

To determine the mechanism underlying APD_20_ prolongation, we measured *I*_Ca,L_, the major depolarizing current in early repolarization.^33^ As illustrated by the representative current traces (Figure 6A) and mean I‐V relations (Figure 6B), *I*_Ca,L_ density was larger in both DMD female and male than in control iPSC‐CMs (female—at voltage steps from 0 mV to −20 mV and male—at 0 mV). No differences were observed in the current activation curve among the three groups (Figure 6C). In agreement with larger *I*_Ca,L_ density, qRT‐PCR analysis demonstrated increased expression of the Ca^2+^ voltage‐gated channel subunit alpha1 C (CACNA1C) in DMD female and male compared to control iPSC‐CMs (Figure 6D).

#### 3.4.4 Differences in the BRV between male and female patients

**Figure 7:**
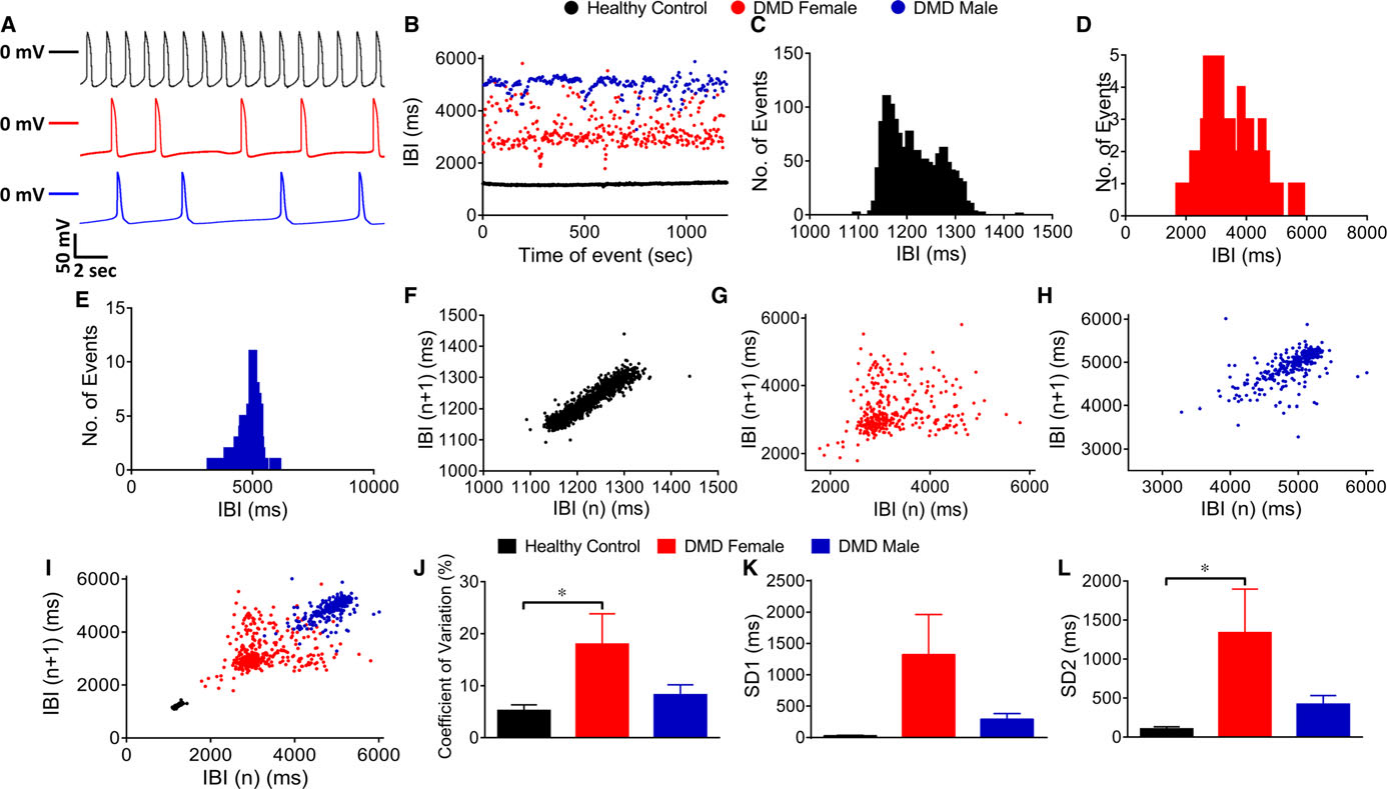
Beat rate variability (BRV) analysis in control and DMD. (A) Representative action potential recordings demonstrating BRV in the different clones. (B) Superimposed representative inter‐beat‐interval (IBI) scatter plots of control, DMD female and male iPSC‐CMs. Representative IBI histograms of control (C), DMD female (D) and DMD male iPSC‐CMs (E). Poincaré plots of control (F), DMD female (G) and DMD male iPSC‐CMs (H). (I) Superimposed Poincaré plots of the three groups. (J) Coefficient of variation (CV) analysis. (K) Standard deviation 1 (SD1) analysis. (L) SD2 analysis. Control n = 11; DMD female, n = 11; DMD male, n = 12. One‐way ANOVA followed by Holm‐Sidak post‐hoc analysis. *P < 0.05

BRV representing the dynamic non‐linear features of automaticity^34^ was analysed in spontaneous action potential recordings (1000‐1800 sec) (Figure 7A). Whereas DMD female iPSC‐CMs displayed more dispersed inter‐beat interval (IBI) scatter plot and histogram than control (Figure 7B‐D), DMD male iPSC‐CMs IBI scatter plot and histogram were similar to control. Representative Poincaré plots, a quantitative measure of the evolvement in time of the BRV, demonstrate the firing pattern abnormalities in DMD female and male iPSC‐CMs compared to the typical ellipsoid‐shaped control Poincaré plot (Figure 7F‐I). Furthermore, coefficient of variation (CV) and SD2, referring to long term IBI variability, were increased in DMD female iPSC‐CMs compared to control (Figure 7J‐L), DMD male iPSC‐CMs CV and SDs were similar to control (Figure 7J‐L). Overall, DMD female iPSC‐CMs displayed increased BRV measures including IBI CV and SD2 Poincaré plot index compared to control iPSC‐CMs. Importantly, similar results were obtained at the network level, from analysis of extracellular electrograms recorded by means of microelectrode array (MEA; Figure S5).

## 4 DISCUSSION

In support of the hypothesis that dystrophin‐mutated iPSC‐CMs exhibit key features of DMD disease, our main findings were: (1) WB analysis showed expression of full length 427 kD dystrophin in control and DMD female iPSC‐CMs, but not in DMD male iPSC‐CMs, confirming that the male patient carries a null allele; (2) XIST and dystrophin expression analyses showed that iPSC generation from female dermal cells is associated with transcriptional reactivation of the inactivated X chromosome either through XCR or erosion of XCI, which is maintained during iPSC‐CM differentiation; (3) DMD female iPSC‐CM population expresses both WT and mutated dystrophin alleles; (4) DMD female and male iPSC‐CMs display arrhythmias and APD prolongation.

### 4.1 Unique genetic features of DMD female iPSC‐CMs population exhibiting mosaic expression of DMD alleles

DMD female carriers are usually unaffected by dystrophin mutations because of skewed XCI preferably silencing the mutated allele.^11^ However, in this study we generated iPSC‐CMs from a heterozygous female manifesting carrier who presented severe adult onset DCM and myopathy. Importantly, this woman carried a highly malignant out‐of‐frame deletion in the 5′ terminal of the DMD gene which caused a severe disease that resulted in teenage death of her son. Genetic analysis demonstrates the DMD female iPSC‐CM population mixed expression of WT dystrophin allele alongside the mutated allele (Figure 1). During reprogramming, X chromosome status is destined to be one of the following three options: (1) conserved inactivation pattern of the originating somatic cell (XaXi); (2) reactivation of the inactive X chromosome (XaXa); (3) erosion of XCI (XaXe).^12^, ^13^, ^14^, ^15^, ^16^, ^17^, ^18^ Considering XIST expression does not sufficiently indicate X chromosome status, and based on the NGS dystrophin expression analyses, a likely possibility is that a mixed population was established during reprogramming and each differentiation.^17^, ^18^ In this scenario, DMD female iPSC‐CMs can be divided to two sub‐populations: (1) cells expressing the WT allele, and (2) cells expressing the mutated allele.^11^ Nonsense mediated decay of mRNA containing premature stop codon, reduces the amount of detectable mutated mRNA in the samples, thus hampering exact quantification of the ratio between these two sub‐populations.^35^ Nevertheless, our genetic analysis faithfully represents the two dystrophin alleles expression ratio in the iPSC‐CM population used for the functional experiments. Furthermore, these novel results display an abnormally increased expression of WT allele in the iPSC‐CM population, implying the first sub‐population (expressing the WT allele) also carries an abnormality compared to control iPSC‐CMs.

### 4.2 Electrophysiological abnormalities in DMD iPSC‐CMs

DMD iPSC‐CMs exhibit a variety of electrophysiological abnormalities divided into four categories: (1) decreased automaticity and altered *I*_f_ density; (2) arrhythmias including DADs and OPPs; (3) prolonged APD and enhanced *I*_Ca,L_ density; (4) increased BRV in DMD female iPSC‐CMs.

#### 4.2.1 Decreased automaticity and altered *I*_f_ density

Contrary to DMD patients who usually manifest increased high heart rate,^32^ DMD iPSC‐CMs display low spontaneous firing rate compared to control. However, in DMD patients, increased heart rate is attributed to elevated sympathetic tone^36^; hence, as this masking effect is absent in DMD iPSC‐CMs, the in vitro study exposes a fundamental pathophysiology of the pacemaker function—attenuated automaticity. To elucidate the mechanism underlying this reduced automaticity in the male and female cardiomyocytes, we recorded *I*_f_, a major pacemaker current.^21^ In agreement with the reduced automaticity in DMD male cardiomyocytes, *I*_f_ density was smaller than control, albeit similar mRNA expression levels of HCNs. In DMD female iPSC‐CMs *I*_f_ density was similar to control, while mRNA expression levels of HCNs were significantly increased. Overall, both DMD female and male iPSC‐CMs demonstrate reduced *I*_f_ density relatively to their respective mRNA expression levels of HCNs. The precise mechanism involved in altered HCN function is yet to be determined. No differences were observed in current activation between DMD and control iPSC‐CMs.

#### 4.2.2 Arrhythmias including DADs and OPPs

In contrast to regular firing pattern in control iPSC‐CMs, 52% and 17% of DMD female and male cardiomyocytes exhibited arrhythmias including DADs and OPPs, respectively. The common cause for DADs is intracellular Ca^2+^overload leading to Na^+^/Ca^2+^ exchanger activation resulting in transient inward depolarizing current (*I*_Ti_) and DADs initiation.^37^ The proposed mechanisms underlying Ca^2+^overload in DMD cardiomyocytes are: (1) damaged sarcolemma enables penetration of extracellular Ca^2+^;^7^ (2) Inflammatory mediators increase the expression of iNOS which binds to and destabilizes the ryanodine receptors (RyRs) located on the sarcoplasmic reticulum (SR), resulting in SR Ca^2+^ leakage into the cytosol.^38^ Notably, leaky RyRs, which can lead to Ca^2+^overload, were reported in *mdx* mice.^39^ Furthermore, *I*_Ca,L_ was increased in DMD iPSC‐CMs compared to control iPSC‐CMs which may contribute to Ca^2+^ overload. The phenomenon of DADs and OPPs in DMD iPSC‐CMs is in agreement with increased Ca^2+^ influx and Ca^2+^ overload, which can contribute to cardiac arrhythmias in DMD patients.^40^

#### 4.2.3 Prolonged APD and enhanced *I*_Ca,L_ density

Importantly, the prolonged APD in female and male DMD cardiomyocytes, potentially associated with fatal arrhythmias,^41^ may account for clinical phenotypes observed in DMD patients, such as long QT interval (LQT).^42^ The ratio between the changes in APD_20_ and APD_90_ strongly implies that most of the prolongation stems from early repolarization phase. As *I*_Ca,L_ is the major depolarizing current during early repolarization,^33^ we measured its density which was higher in both DMD female and male iPSC‐CMs. These results are consistent with previous findings of enhanced *I*_Ca,L_ in adult cardiomyocytes of *mdx* mice.^43^ Furthermore, CACNA1C expression was increased in both DMD female and male iPSC‐CMs compared to control.

#### 4.2.4 Increased BRV in DMD female iPSC‐CMs

DMD female (but not male) iPSC‐CMs displayed increased BRV measures compared to control cardiomyocytes. While further investigation is required to determine the contribution of the mixed dystrophin expression to this finding, it is likely that heterogeneity of the cardiomyocytes expressing either WT or mutated dystrophin alleles, contributes to increased BRV indices in action potential recordings from iPSC‐CM clusters.

### 4.3 Advantages of DMD iPSC‐CMs over the *mdx* mouse model

The common murine model for DMD research is the *mdx* mouse which carries a point mutation in exon 23 of the dystrophin gene.^43^ However, the similarity of *mdx* hearts to human DMD hearts is debated; while mice initially develop hypertrophic cardiomyopathy and only at a later stage contractile dysfunction and systolic heart failure, human cardiomyopathy is characterized primarily by ventricular dilatation and reduced systolic function.^44^ The use of patients’ iPSC‐CMs has been previously demonstrated as valid cellular in vitro model for various genetic heart diseases including DMD,^45^, ^46^, ^47^, ^48^ as well for LQT syndrome,^41^ and *PRKAG2* myopathy.^49^ Furthermore, iPSC‐CMs are an accessible human cellular model which also enables exclusion of in vivo masking factors such as the influence of the autonomous nervous system.

### 4.4 Summary and conclusions

In conclusion, this study demonstrates that loss of dystrophin in patients’ iPSC‐CMs is sufficient to cause electrophysiological abnormalities including low spontaneous firing rate, arrhythmogenic firing patterns, abnormal action potential parameters, increased BRV and altered pacemaker currents density. Importantly, this is the first study demonstrating the impact of mixed dystrophin expression pattern on abnormal characteristics and functional abnormalities of DMD iPSC‐CMs generated from a DMD female manifesting carrier. These results may suggest greater predisposition to arrhythmias in early stages of DMD cardiomyopathy in females. Further studies are therefore required to characterize the natural history and define the arrhythmic risk in female patients with DMD mutation. This study also has implications for genetic therapies aimed at restoring dystrophin expression by gene editing or gene therapy; if genetic correction is incomplete, a subset of dystrophin deficient myocytes may increase electrophysiological heterogeneity which could be a substrate for arrythmogenesis.

## CONFLICT OF INTEREST

The authors have nothing to declare.

## References

[1] Hoffman EP , Brown RH , Kunkel LM . Dystrophin: the protein product of the Duchenne muscular dystrophy locus. Cell. 1987;51:919‐928. 3319190 10.1016/0092-8674(87)90579-4

[2] Parsons EP , Clarke AJ , Hood K , et al. Newborn screening for Duchenne muscular dystrophy: a psychosocial study. Arch Dis Child Fetal Neonatal Ed. 2002;86:91‐95. 10.1136/fn.86.2.F91 PMC1721374 11882550

[3] Dooley J , Gordon KE , Dodds L , et al. Duchenne muscular dystrophy: a 30‐year population‐based incidence study. Clin. Pediatr. (Phila). 2010;49:177‐179. 20080524 10.1177/0009922809347777

[4] Papa R , Madia F , Bartolomeo D , et al. Genetic and early clinical manifestations of females heterozygous for Duchenne/Becker muscular dystrophy. Pediatr. Neurol. 2016;55:58‐63. 26718981 10.1016/j.pediatrneurol.2015.11.004

[5] Hoogerwaard EM , Bakker E , Ippel PF , et al. Signs and symptoms of Duchenne muscular dystrophy and Becker muscular dystrophy among carriers in The Netherlands: a cohort study. Lancet (London, England). 1999;353:2116‐2119. 10.1016/s0140-6736(98)10028-4 10382696

[6] Soltanzadeh P , Friez MJ , Dunn D , et al. Clinical and genetic characterization of manifesting carriers of DMD mutations. Neuromuscul. Disord. 2010;20:499‐504. 20630757 10.1016/j.nmd.2010.05.010 PMC2944769

[7] Millay DP , Goonasekera SA , Sargent MA , et al. Calcium influx is sufficient to induce muscular dystrophy through a TRPC‐dependent mechanism. Proc. Natl. Acad. Sci. U.S.A. 2009;106:19023‐19028. 19864620 10.1073/pnas.0906591106 PMC2776441

[8] Brenman JE , Chao DS , Gee SH , et al. Interaction of nitric oxide synthase with the postsynaptic density protein PSD‐95 and alpha1‐syntrophin mediated by PDZ domains. Cell. 1996;84:757‐767. 8625413 10.1016/s0092-8674(00)81053-3

[9] Desguerre I , Mayer M , Leturcq F , et al. Endomysial fibrosis in Duchenne muscular dystrophy: a marker of poor outcome associated with macrophage alternative activation. J. Neuropathol. Exp. Neurol. 2009;68:762‐773. 19535995 10.1097/NEN.0b013e3181aa31c2

[10] McNally EM , Kaltman JR , Benson DW , et al. Contemporary cardiac issues in Duchenne muscular dystrophy. Working Group of the National Heart, Lung, and Blood Institute in collaboration with Parent Project Muscular Dystrophy. Circulation. 2015;131:1590‐1598. 25940966 10.1161/CIRCULATIONAHA.114.015151 PMC4573596

[11] Viggiano E , Ergoli M , Picillo E , et al. Determining the role of skewed X‐chromosome inactivation in developing muscle symptoms in carriers of Duchenne muscular dystrophy. Hum Genet. 2016;135:685‐698. 27098336 10.1007/s00439-016-1666-6

[12] Yoshida S , Nakanishi C , Okada H , et al. Characteristics of induced pluripotent stem cells from clinically divergent female monozygotic twins with Danon disease. J. Mol. Cell. Cardiol. 2018;114:234‐242. 29175505 10.1016/j.yjmcc.2017.11.019

[13] Miyagoe‐Suzuki Y , Nishiyama T , Nakamura M , et al. Induction of pluripotent stem cells from a manifesting carrier of Duchenne muscular dystrophy and characterization of their X‐inactivation status. Stem Cells Int. 2017;2017:1‐9. 10.1155/2017/7906843 PMC5405591 28491099

[14] Lyu C , Shen J , Zhang J , et al. The state of skewed X chromosome inactivation is retained in the induced pluripotent stem cells from a female with hemophilia B. Stem Cells Dev. 2017;26:1003‐1011. 28401797 10.1089/scd.2016.0323

[15] Barakat TS , Ghazvini M , de Hoon B , et al. Stable X chromosome reactivation in female human induced pluripotent stem cells. Stem Cell Reports. 2015;4:199‐208. 25640760 10.1016/j.stemcr.2014.12.012 PMC4325229

[16] Cheung A , Horvath LM , Carrel L , et al. X‐chromosome inactivation in Rett syndrome human induced pluripotent stem cells. Front. Psychiatry. 2012;3:24. 22470355 10.3389/fpsyt.2012.00024 PMC3311266

[17] Dandulakis MG , Meganathan K , Kroll KL , et al. Complexities of X chromosome inactivation status in female human induced pluripotent stem cells‐a brief review and scientific update for autism research. J. Neurodev. Disord. 2016;8:22. 27303449 10.1186/s11689-016-9155-8 PMC4907282

[18] Lessing D , Lee JT . X Chromosome inactivation and epigenetic responses to cellular reprogramming. Annu. Rev. Genomics Hum. Genet. 2013;14:85‐110. 23662665 10.1146/annurev-genom-091212-153530

[19] Eisen B , Ben Jehuda R , Cuttitta AJ , et al. Generation of Duchenne muscular dystrophy patient‐specific induced pluripotent stem cell line lacking exons 45–50 of the dystrophin gene (IITi001‐A). Stem Cell Res. 2018;29:111‐114. 29653394 10.1016/j.scr.2018.03.023 PMC5999581

[20] Lian X , Zhang J , Azarin SM , et al. Directed cardiomyocyte differentiation from human pluripotent stem cells by modulating Wnt/β‐catenin signaling under fully defined conditions. Nat. Protoc. 2013;8:162‐175. 23257984 10.1038/nprot.2012.150 PMC3612968

[21] DiFrancesco D . The role of the funny current in pacemaker activity. Circ. Res. 2010;106:434‐446. 20167941 10.1161/CIRCRESAHA.109.208041

[22] Hwang HS , Kryshtal DO , Feaster TK , et al. Comparable calcium handling of human iPSC‐derived cardiomyocytes generated by multiple laboratories. J. Mol. Cell. Cardiol. 2015;85:79‐88. 25982839 10.1016/j.yjmcc.2015.05.003 PMC4530041

[23] Marchetto M , Carromeu C , Acab A , et al. A model for neural development and treatment of Rett syndrome using human induced pluripotent stem cells. Cell. 2010;143:527‐539. 21074045 10.1016/j.cell.2010.10.016 PMC3003590

[24] Vallot C , Ouimette J‐F , Makhlouf M , et al. Erosion of X chromosome inactivation in human pluripotent cells initiates with XACT coating and depends on a specific heterochromatin landscape. Cell Stem Cell. 2015;16:533‐546. 25921272 10.1016/j.stem.2015.03.016

[25] Mekhoubad S , Bock C , de Boer AS , et al. Erosion of dosage compensation impacts human iPSC disease modeling. Cell Stem Cell. 2012;10:595‐609. 22560080 10.1016/j.stem.2012.02.014 PMC3603710

[26] Tchieu J , Kuoy E , Chin MH , et al. Female human iPSCs retain an inactive X chromosome. Cell Stem Cell. 2010;7:329‐342. 20727844 10.1016/j.stem.2010.06.024 PMC2935700

[27] Pegoraro E , Schimke RN , Garcia C , et al. Genetic and biochemical normalization in female carriers of Duchenne muscular dystrophy: evidence for failure of dystrophin production in dystrophin‐competent myonuclei. Neurology. 1995;45:677‐690. 7723955 10.1212/wnl.45.4.677

[28] Khan SA , Audergon P , Payer B . X‐chromosome activity in naive human pluripotent stem cells‐are we there yet? Stem Cell Investig. 2017;4:54. 10.21037/sci.2017.06.03 PMC5503910 28725650

[29] Liang G , Zhang Y . Genetic and epigenetic variations in iPSCs: potential causes and implications for application. Cell Stem Cell. 2013;13:149‐159. 23910082 10.1016/j.stem.2013.07.001 PMC3760008

[30] Geens M , Seriola A , Barbé L , et al. Female human pluripotent stem cells rapidly lose X chromosome inactivation marks and progress to a skewed methylation pattern during culture. Mol. Hum. Reprod. 2016;22:285‐298. 26786180 10.1093/molehr/gaw004

[31] Takami Y , Takeshima Y , Awano H , et al. High incidence of electrocardiogram abnormalities in young patients with Duchenne muscular dystrophy. Pediatr. Neurol. 2008;39:399‐403. 19027585 10.1016/j.pediatrneurol.2008.08.006

[32] Rajdev A , Groh WJ . Arrhythmias in the muscular dystrophies. Card. Electrophysiol. Clin. 2015;7:303‐308. 26002394 10.1016/j.ccep.2015.03.011 PMC4441951

[33] Grant AO . Cardiac ion channels. Circ. Arrhythmia Electrophysiol. 2009;2:185‐194. 10.1161/CIRCEP.108.789081 19808464

[34] Ben‐Ari M , Schick R , Barad L , et al. From beat rate variability in induced pluripotent stem cell‐derived pacemaker cells to heart rate variability in human subjects. Heart Rhythm. 2014;11:1808‐1818. 25052725 10.1016/j.hrthm.2014.05.037 PMC4283811

[35] Linde L , Boelz S , Nissim‐Rafinia M , et al. Nonsense‐mediated mRNA decay affects nonsense transcript levels and governs response of cystic fibrosis patients to gentamicin. J. Clin. Invest. 2007;117:683‐692. 17290305 10.1172/JCI28523 PMC1783999

[36] Dhargave P , Nalini A , Abhishekh HA , et al. Assessment of cardiac autonomic function in patients with Duchenne muscular dystrophy using short term heart rate variability measures. Eur. J. Paediatr. Neurol. 2014;18:317‐320. 24445161 10.1016/j.ejpn.2013.12.009

[37] Verkerk AO , Veldkamp MW , Baartscheer A , et al. Ionic mechanism of delayed afterdepolarizations in ventricular cells isolated from human end‐stage failing hearts. Circulation. 2001;104:2728‐2733. 11723027 10.1161/hc4701.099577

[38] Allen DG , Whitehead NP , Froehner SC . Absence of dystrophin disrupts skeletal muscle signaling: roles of Ca2+, reactive oxygen species, and nitric oxide in the development of muscular dystrophy. Physiol. Rev. 2016;96:253‐305. 26676145 10.1152/physrev.00007.2015 PMC4698395

[39] Fauconnier J , Thireau J , Reiken S , et al. Leaky RyR2 trigger ventricular arrhythmias in Duchenne muscular dystrophy. Proc. Natl. Acad. Sci. U.S.A. 2010;107:1559‐1564. 20080623 10.1073/pnas.0908540107 PMC2824377

[40] Chiang DY , Allen HD , Kim JJ , et al. Relation of cardiac dysfunction to rhythm abnormalities in patients with Duchenne or Becker muscular dystrophies. Am. J. Cardiol. 2016;117:1349‐1354. 26952271 10.1016/j.amjcard.2016.01.031

[41] Itzhaki I , Maizels L , Huber I , et al. Modelling the long QT syndrome with induced pluripotent stem cells. Nature. 2011;471. 10.1038/nature09747 21240260

[42] Thrush PT , Allen HD , Viollet L , et al. Re‐examination of the electrocardiogram in boys with Duchenne muscular dystrophy and correlation with its dilated cardiomyopathy. Am. J. Cardiol. 2009;103:262‐265. 19121448 10.1016/j.amjcard.2008.08.064

[43] Koenig X , Rubi L , Obermair GJ , et al. Enhanced currents through L‐type calcium channels in cardiomyocytes disturb the electrophysiology of the dystrophic heart. Am. J. Physiol. Circ. Physiol. 2014;306:564‐573. 10.1152/ajpheart.00441.2013 PMC4892346 24337461

[44] Khouzami L , Bourin M‐C , Christov C , et al. Delayed cardiomyopathy in dystrophin deficient mdx mice relies on intrinsic glutathione resource. Am. J. Pathol. 2010;177:1356‐1364. 20696779 10.2353/ajpath.2010.090479 PMC2928968

[45] Hashimoto A , Naito AT , Lee J‐K , et al. Generation of induced pluripotent stem cells from patients with Duchenne muscular dystrophy and their induction to cardiomyocytes. Int. Heart J. 2016;57:112‐117. 26673445 10.1536/ihj.15-376

[46] Guan X , Mack DL , Moreno CM , et al. Dystrophin‐deficient cardiomyocytes derived from human urine: New biologic reagents for drug discovery. Stem Cell Res. 2014;12:467‐480. 24434629 10.1016/j.scr.2013.12.004 PMC3966181

[47] Dick E , Kalra S , Anderson D , et al. Exon skipping and gene transfer restore dystrophin expression in human induced pluripotent stem cells‐cardiomyocytes harboring *DMD* mutations. Stem Cells Dev. 2013;22:2714‐2724. 23829870 10.1089/scd.2013.0135 PMC3787465

[48] Lin B , Li Y , Han L , et al. Modeling and study of the mechanism of dilated cardiomyopathy using induced pluripotent stem cells derived from individuals with Duchenne muscular dystrophy. Dis. Model. Mech. 2015;8:457‐466. 25791035 10.1242/dmm.019505 PMC4415895

[49] Ben Jehuda R , Eisen B , Shemer Y , et al. CRISPR correction of the PRKAG2 gene mutation in the patient’s induced pluripotent stem cell‐derived cardiomyocytes eliminates electrophysiological and structural abnormalities. Hear. Rhythm. 2018;15:267‐276. 10.1016/j.hrthm.2017.09.024 28917552

