# Supplementary figures and images for: Electrophysiological abnormalities in induced pluripotent stem cell‐derived cardiomyocytes generated from Duchenne muscular dystrophy patients
Source: J Cell Mol Med. 2019 Jan 8;23(3):2125–35. doi: 10.1111/jcmm.14124 (PMC6378185; doi:10.1111/jcmm.14124)

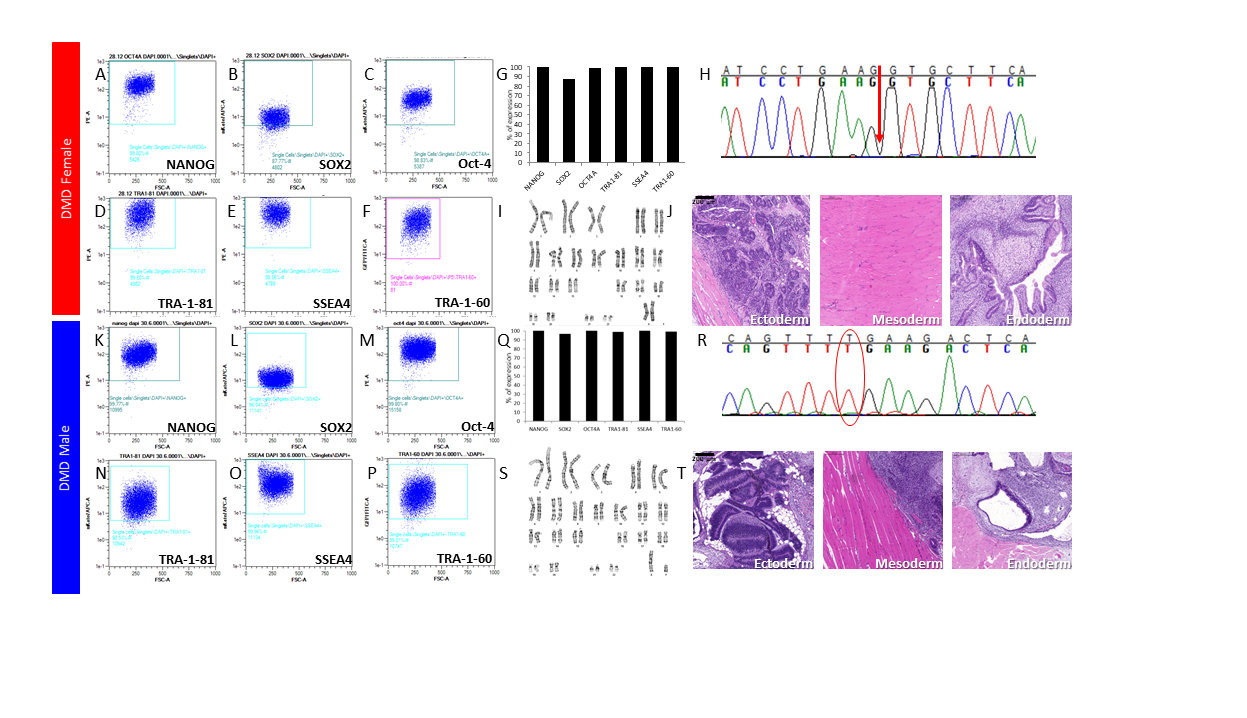

Supplement: Supplementary file 1 [file JCMM-23-2125-s001.tif]

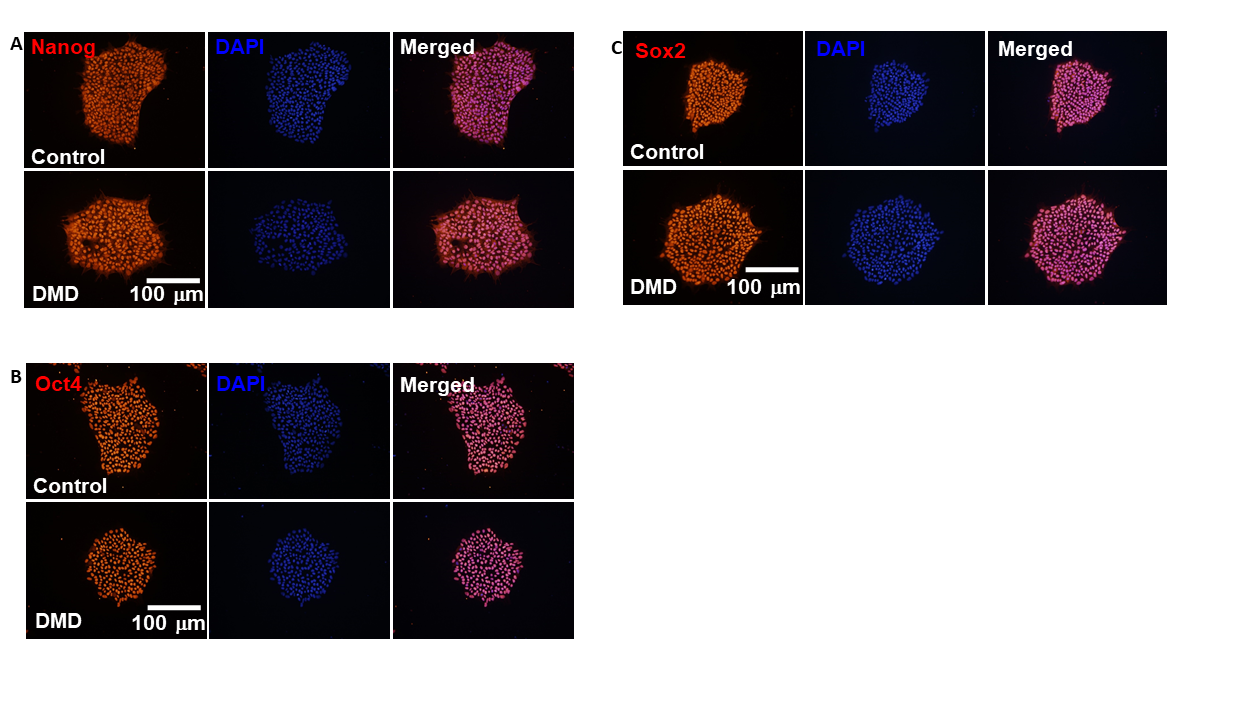

Supplement: Supplementary file 2 [file JCMM-23-2125-s002.tif]

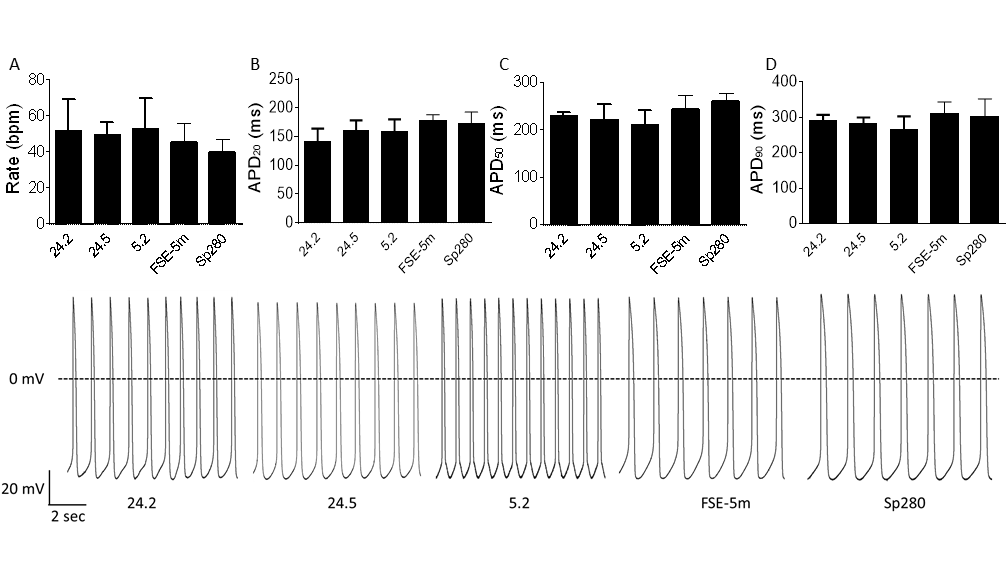

Supplement: Supplementary file 3 [file JCMM-23-2125-s003.tif]

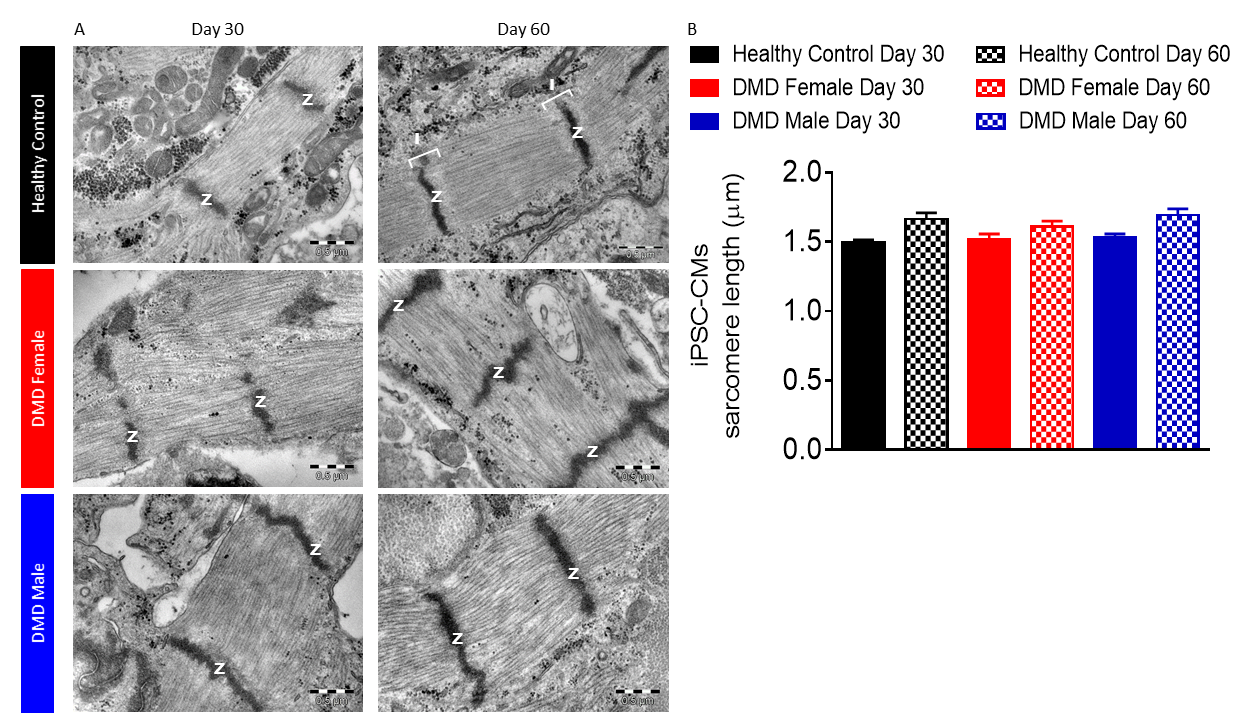

Supplement: Supplementary file 4 [file JCMM-23-2125-s004.tif]

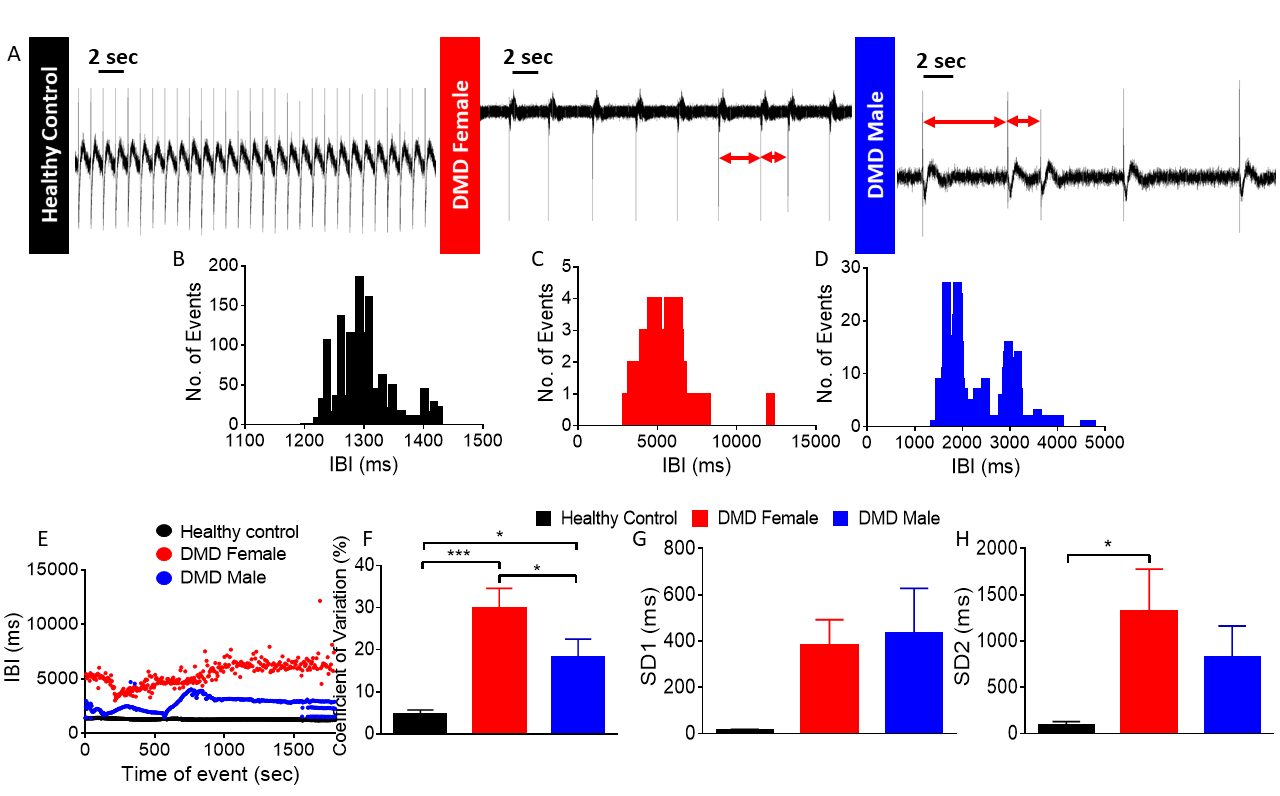

Supplement: Supplementary file 5 [file JCMM-23-2125-s005.tif]
