# Supplementary material for: Electrophysiological abnormalities in induced pluripotent stem cell‐derived cardiomyocytes generated from Duchenne muscular dystrophy patients
Source: J Cell Mol Med. 2019 Jan 8;23(3):2125–35. doi: 10.1111/jcmm.14124 (PMC6378185; doi:10.1111/jcmm.14124)
Supplement: Supplementary file 6 [file JCMM-23-2125-s006.docx]

**Electrophysiological abnormalities in induced pluripotent stem cell-derived cardiomyocytes generated from Duchenne muscular dystrophy patients**

**Short title: Electrophysiological abnormalities in DMD iPSC-CMs**

Binyamin Eisen, MSc,^a,^*; Ronen Ben Jehuda, MSc,^a, b,^*; Ashley J. Cuttitta, MSc,^c^; Lucy N. Mekies, MSc,^a^; Yuval Shemer, MSc,^a^; Polina Baskin, BSc,^a^; Irina Reiter, MSc,^a^; Dov Freimark, MD,^d,e^; Mihaela Gherghiceanu, MD, PhD,^f^; Lorenzo Monserrat, MD, PhD,^g^; Michaela Scherr, PhD,^h^; Denise Hilfiker-Kleiner, PhD,^i^; Michael Arad, MD,^d,e,#^; Daniel E. Michele, PhD,^c,#^; Ofer Binah, PhD,^a,#^.

^a^Department of Physiology, Biophysics and Systems Biology, Rappaport Faculty of Medicine, Technion – Israel Institute of Technology, Haifa, Israel. ^b^Department of Biotechnology, Technion – Israel Institute of Technology, Haifa, Israel. ^c^Department of Molecular and Integrative Physiology, University of Michigan, Ann Arbor, Michigan, USA. ^d^Leviev Heart Center, Sheba Medical Center, Ramat Gan, Israel. ^e^Sackler Faculty of Medicine, Tel Aviv University, Tel Aviv, Israel. ^f^Victor Babes National Institute of Pathology, Bucharest, Romania. ^g^Health in Code, A Corunna, Spain. ^h^Department of Hematology, Hemostasis, Oncology and Stem Cell Transplantation, Hannover Medical School, Germany. ^i^Department of Cardiology and Angiology, Hannover Medical School, Hannover, Germany.

*These authors contributed equally to this study.

^#^These authors contributed equally to this study.

Corresponding authors

Ofer Binah, PhD

Cardiac Research Laboratory

Department of Physiology, Biophysics and Systems Biology

Rappaport Faculty of Medicine

Technion – Israel Institute of Technology

1 Efron St.

Haifa 3525422, Israel

Email: [binah@tx.technion.ac.il](mailto:binah@tx.technion.ac.il)

Daniel Michele, PhD

Department of Molecular and Integrative Physiology

Department of Internal Medicine

University of Michigan

North Campus Research Complex

2800 Plymouth Road, Bldg. 26, Room 207S

Ann Arbor, MI 48109-2800, USA

Email: [dmichele@umich.edu](mailto:dmichele@umich.edu)

**Supplement Figure legends**

Figure S1

DMD iPSC clones' pluripotency markers fluorescence-activated cell sorting (FACS) analysis, Sanger sequencing, karyotypes and teratomas. (A-G, K-R) FACS analysis demonstrated the presence of pluripotent markers (NANOG, Sox2, Oct-4, TRA1-81, SSEA4, and TRA1-60) in both DMD iPSC clones. (H, S). Sanger sequencing of both DMD iPSC clones demonstrated their respective genetic mutations corresponding to the donors. (I, T) Karyotype results of both DMD iPSC clones revealed the cells maintained chromosomal integrity during reprograming. (J, U) Teratomas generated from the two DMD iPSC clones included cell types of the 3 germ layers – ectoderm, mesoderm and endoderm.

Figure S2

Control and DMD male iPSCs express pluripotency associated transcription factors (TF) Nanog, Oct4, and Sox2. A-C show immunofluorescence (IF) staining for Nanog, Oct3 and Sox2, respectively. All colonies were also stained with DAPI. Immunofluorescence staining demonstrated that control and DMD iPSCs express 3 essential pluripotency transcription factors (TFs) validating the FACS analysis in Fig. S1.

Figure S3

Comparison of action potential parameters of the 5 control clones. (A) Rate; (B-D) Action potential duration at 20/50/90% of repolarization (APD_20/50/90_). One-way ANOVA followed by Holm-Sidak *post-hoc* analysis. P>0.85 for all comparisons.

Figure S4

Transmission electron microscopy (TEM) analysis of DMD and control iPSC-CMs. (A) TEM analysis of 30 and 60 days old iPSC-CMs demonstrated no significant ultrastructural difference between DMD and control iPSC-CMs. (B) Sarcomere length of DMD and control iPSC-CMs was not significantly different in both 30 and 60 days old cells. Control n=25; DMD female, n=25; DMD male, n=25. One-way ANOVA followed by Holm-Sidak *post-hoc* analysis.

Figure S5

Beat rate variability (BRV) analysis in control and DMD at the network level. (A) Representative extracellular recordings demonstrating BRV in the three groups; dissimilar inter-beat-intervals (IBI) are indicated by red arrows. Representative IBI histograms of control (B), DMD female (C) and DMD male iPSC-CMs (D). (E) Superimposed representative IBI scatter plots of control, DMD female and male iPSC-CMs. (F) Coefficient of variation (CV) analysis. (G) standard deviation 1 (SD1) analysis. (H) SD2 analysis. Control n=6; DMD female, n=3; DMD male, n=5. One-way ANOVA followed by Holm-Sidak *post-hoc* analysis. *p<0.05, **p<0.01.

**Supplemental Material**

Methods

**The source of the DMD-mutated patients' dermal fibroblasts**

Dermal biopsies were obtained from AN, a 50-year-old DMD manifesting female patient carrying a deletion of exons 8-12 (ex.8_12del) and GF, a 32-year-old DMD male patient carrying a substitution of cytosine to thymine (c.5899C>T) constituting a premature stop codon. The donors signed a consent form according to approval #7603-09-SMC by the Helsinki Committee for Experiments on Human Subjects at Sheba Medical Center, Ramat Gan, Israel. Four more clones (see details below) were characterized earlier.

**Generation of induced pluripotent stem cells (iPSCs) from the patient and healthy volunteers**

iPSCs were generated from the patients' dermal fibroblasts using Sendai virus CytoTune-iPS 2.0 Sendai Reprogramming Kit, #A16517 (Thermo Fisher, Waltham, MA, USA) for the transfection of Yamanaka’s 4 factors: Oct-4, KLF4, c-Myc, SOX2 as previously described [1]. As control, we used the following clones: clones 24.2 and 24.5 (generated from a 42-year old female) as previously characterized and described [2]; clone 5.2 (generated from a 25-year old male) as previously characterized and described [3]; clone FSE-5m (generated from neonatal foreskin fibroblasts) as previously characterized and described [4]; clone Sp280 (generated from a 50-year old male) as previously characterized and described [5]. All five clones were used for analyses and comparisons with the diseased clones.

**Karyotype analysis**

Karyotype analysis was conducted according to standard procedures as previously described [3].

**Teratoma Formation**

To verify the iPSCs differentiation capacity *in vivo*, iPSCs colonies from two 6-well plates were detached using 1 mg/ml type IV collagenase, washed 3 times in PBS and injected into the thigh muscle of severe combined immunodeficient (SCID) mice. Teratomas were observed 8-12 weeks after injection, and images were obtained from formalin-fixed (4%) and paraffin-embedded teratoma sections stained with hematoxylin and eosin (H&E).

**Genotyping**

For the male patient's iPSCs, genomic DNA was isolated from fibroblasts and iPSCs using QIAGEN DNeasy Blood & Tissue Kit (QIAGEN, Hilden, Germany). PCR was performed to the dystrophin gene using the primers listed in the primers table resulting in a product of 500 base pairs in length. Next, the PCR product was cleaned using QIAquick PCR Purification Kit (QIAGEN, Hilden, Germany), followed by Sanger sequencing for the PCR product containing the mutation area within the dystrophin gene. Subsequently, we confirmed the missense point mutation (substitution of Cytosine to Thymine) in exon 44 leading to the replacement of arginine with a pre-mature stop codon. For the female patient's iPSCs, RNA was produced using High-Capacity cDNA Reverse Transcription Kit (Thermo Fisher, Waltham, MA, USA) and cDNA was generated using QIAGEN RNeasy Kit (QIAGEN, Hilden, Germany); subsequently, PCR and Sanger sequencing was performed using the primers listed in Table 1.

Table 1: Primers table

|  |  | Forward | Reverse |
| --- | --- | --- | --- |
| Amplification | DMD-Male | AATCCCAAGACACCAGAGGA | TGGCAAACCCACGTATGTGT |
| Amplification | DMD-Female-cDNA RT-PCR Ex.7-13 | AGTCAGCCACACAACGACTG | TCTCACTCACATGGTGGTGG |
| Reverse transcription | DMD-RT16 |  | CCGTCTTCTGGGTCACTGAC |
| Amplification | DMD-RT6-14 | GGCTTTGAATGCTCTCATCC | CGTTGCCATTTGAGAAGGAT |
| Amplification | DMD-RT8-12 | AAGTGGAAATGTTGCCAAGG | AGGCTCTTCCTCCATTTTCC |

**Differentiation into cardiomyocytes**

iPSC-CMs were generated according to the directed differentiation by modulating Wnt/β-catenin signaling as previously described [6]. Briefly, iPSCs were cultured on Matrigel (GFR, BD Biosciences, Franklin Lakes, NJ, USA) coated 6-well plates in mTeSR1 medium ([Stemcell Technologies](http://www.stemcell.com/en/products/all-products/mtesr1.aspx), Vancouver, Canada) for 5-6 days. To initiate differentiation, cells were incubated with 1 ml/well Versene solution (Invitrogen, Life Technologies, Woburn, [MA, USA](http://en.wikipedia.org/wiki/Waltham,_Massachusetts)) at 37^o^C for 8 minutes and seeded on Matrigel coated plate at 8.5x10^6^/12 well plate density in mTeSR1 medium supplemented with 5 μmol/l ROCK inhibitor ([Cayman Chemical](https://www.caymanchem.com/catalog/10005583), Ann Arbor, MI, USA). The medium was replaced daily, and after 2 days when the monolayer of cells reached 100% confluence, the medium was changed to RPMI supplemented with B27 minus insulin (Invitrogen, Life Technologies, Woburn, [MA, USA](http://en.wikipedia.org/wiki/Waltham,_Massachusetts)) containing 8 or 10 μmol/l CHIR99021 and this day was labeled as day 1 of differentiation. On the next day (day 2 of differentiation), the medium was changed to RPMI supplemented with B27 minus insulin. On the 4^th^ day, the medium was changed to RPMI supplemented with B27 minus insulin, containing 5 or 10 μmol/l of IWP-4 or 2. On the 6^th^ day, the medium was changed to RPMI supplemented with B27 minus insulin. Finally, from the 8^th^ day onwards, the medium was changed to RPMI supplemented with B27 complete supplement (Invitrogen, Life Technologies, Woburn, [MA, USA](http://en.wikipedia.org/wiki/Waltham,_Massachusetts)).

**RNA analysis of the *dystrophin* gene**

RNA extraction

RNA extraction from 10^6^ cells was carried out using the “ReliaPrep™ RNA Cell Miniprep System Kit” (Promega, Fitchburg, WI, USA) according to the manufacturer’s instructions.

RNAseq methods to evaluate the relative expression levels of the dystrophin alleles

Since DMD expression levels are relatively low, two enrichment approaches were used to determine the relative expression levels of both the mutant and WT DMD alleles in iPSC-CMs. First, cDNAs were enriched in genes involved in cardiovascular disease using a custom capture probe (Agilent, Santa Clara, CA, USA) library designed against the coding region of 213 genes. Extracted RNAs were processed using the “SureSelect Strand-Specific RNA Library Prep for Illumina Multiplexed Sequencing mRNA Library Preparation Protocol” (Agilent, Santa Clara, CA, USA) with modifications. Briefly, RNAs were processed up to the amplification and indexing step following the instructions. From this point, hybridization and post-hybridization steps were carried out using the “Sure Select XT target enrichment system for Ilumina Paired end sequencing library protocol” (Agilent, Santa Clara, CA, USA). The resulting libraries were sequenced on a 1500 Illumina Hi-Seq at 2 x 100 (Ilumina, San Diego, CA, USA). Second, a RT-PCR strategy specifically targeted against the region of interest was used. Total RNA from iPSC-CMs were DNase I treated and first strand cDNA was transcribed using the “AccuScript Reverse Transcriptase” (Agilent, Santa Clara, CA, USA), primed with an antisense oligonucleotide annealing at DMD exon 16. RT-PCR was carried out using pairs of primers annealing at exons 6 and 14 (flanking the deletion) or at exons 8-12 (within the deleted region). All nucleotide sequences are shown in Table 1. PCR products were fragmented using a Covaris E220 ultrasonicator and processed using the NEXTflex™ Rapid DNA Sequencing Kit (Bioo Scientific, Austin, TX, USA), following the manufacturer’s instructions. The resulting libraries were sequenced on a 1500 Illumina Hi-Seq at 2 x 100.

**RNA isolation, and TaqMan analyses for XIST RNA**

Total RNA from cells was prepared using Trizol (Invitrogen). For qRT-PCR of mRNAs, cDNA synthesis was performed with 1 µg of total RNA digested with DNaseI and subjected to TaqMan-based (Applied Biosystems) gene expression profiling following the manufacturer’s protocol. Primer/probe assays for human XIST (Hs01079824_m1), and ß-Actin (Hs99999903_m1) were purchased from Applied Biosystems. ß-Actin served as an internal control. Real-time PCR was performed using an ABI7500 cycler (Applied Biosystems).

**qPCR of Dystrophin Glycoprotein Complex genes during small molecule differentiation into iPSC-CMs**

Patient derived iPSCs and iPSC-CMs were washed with DPBS and scraped up from cell culture dishes using a cell lifter (Corning 3008) in cold TRIzol reagent (Life Technologies, 15596026) at days 0, 7, 14, 21, and 30 of the GiWi small molecule differentiation protocol described in Lian et al. Nature Protocols Vol 8 No 1 2013. Samples were immediately frozen in liquid nitrogen and stored in TRIzol at -80ºC until use. At time of RNA extraction, the samples were thawed on ice and vortexed for 10 seconds for further lysing. RNA was extracted per TRIzol manufacturer's instructions. The samples were treated with DNase and further purified using RNeasy Mini Kit (Qiagen, 74104) RNA cleanup protocol. All RNA samples had measured 260/280 between 2-2.2. In addition, 0.2-1 µg of the RNA was run on a denaturing gel to confirm the presence of RNA in each sample. Using 0.3 μg of total RNA, cDNA was made using a High Capacity cDNA Reverse Transcription Kit with RNase Inhibitor (Applied Biosystems, 4374966). A quantitative PCR experiment was performed using iTaq Universal SYBR Green Supermix (Bio -Rad, 172-5121) with a Step One Plus Real-Time PCR System (Applied Biosystems) using primers as listed in Table 3. A comparative Ct method was used to quantify the target gene expression. All data is normalized to day 0.

Table 3: Primers table

| **Gene** | **qPCR Primers** | |
| --- | --- | --- |
|  | **Forward Sequence** | **Reverse Sequence** |
| DMD-N | 5-CAC TGG CAG GTC AAA AAT GTA ATG-3 | 5-TGG CCT ATG ACT ATG GAT GAG AGC-3 |
| DMD-C | 5-TGA CCA CTA TTT ATG ACC GCC TG-3 | 5-CTC CCT GTT CGT CCC GTA TC-3 |
| UTRN | 5-CTC GCC TTT AAT GCT GTC CTC CAC-3 | 5-GCA CTT CCT CCA ACG CAA TCT GA-3 |
| SGCG | 5-CCT GTC TGT GGC CGG TGT GA-3 | 5-GCG TTT ACT TCC CAT CCA CGC TGC-3 |

**Reverse Transcription qPCR**

RNA extraction was performed using PureLink RNA mini kit (Thermo Fisher, Waltham, MA, USA). Subsequently, cDNA was produced using High-Capacity cDNA Reverse Transcription Kit (Thermo Fisher, Waltham, MA, USA). Finally, RT-qPCR TaqMan was performed using the following catalog primers (Thermo Fisher, Waltham, MA, USA): GAPDH (Hs99999905_m1) – as housekeeping gene, HCN2 (Hs00606903_m1), HCN4 (Hs00975492_m1), CACNA1C (Hs00167681_m1); with TaqMan Fast Advanced Master Mix – StepOnePlus (Thermo Fisher, Waltham, MA, USA). Expression analysis was done using StepOne Software (Thermo Fisher, Waltham, MA, USA).

Bioinformatics

Primary analyses, including base calling, read filtering and demultiplexing were performed according to the standard Illumina processing pipeline (CASAVA 1.8.2). Sequence read pairs were mapped to the human genome assembly GRCh37 by TopHat v2.1.0 [7] using a slightly modified version of Refseq (April 19, 2017) as a set of known gene/transcript models. The Refseq modification consisted on the addition of a new transcript model that was build using the WT muscular transcript model (NM_004006) as a template but modified properly to reflect the new splicing event generated due to the deletion of exons 8-12 (ex.8_12del). Transcript abundance were estimated by Cufflinks v2.2.1 [8]. Expression values (FPKM) were normalized by library size and corrected by cell line library enrichment factor.

**Flow Cytometry**

DMD iPSCs were grown to confluency, washed with HBSS, dissociated with 0.25% trypsin-EDTA, resuspended in EB20 media, and centrifuged at 1000 rpm for 5 minutes. Pelleted cells were resuspended in cold 3% Paraformaldehyde for 10 minutes to fix. Next, cells were resuspended in cold PBS-T with 3% BSA and incubated on ice for 15 minutes. The cell suspension was centrifuged again at 1000 rpm for 5 minutes and then the supernatant aspirated. Cells were divided six ways and resuspended in antibodies labeling six essential pluripotency makers Nanog (Miltenyi Biotec, 130-105-080), Oct3/4-Isoform A (Miltenyi Biotec, 130-105-606), Sox2 (Miltenyi Biotec, 130-104-995), TRA-1-81 (Miltenyi Biotec, 130-101-427), SSEA-4 (Miltenyi Biotec, 130-100-635), or TRA-1-60 (Miltenyi Biotec, 130-106-872). Cells were incubated with antibodies on ice for 40 minutes using a 1:10 dilution in PBS-T with 3% BSA. Following antibody labeling, cells were incubated for 5 minutes on ice with a 1:1000 DAPI dilution. After labeling, MACS buffer (Miltenyi Biotec, 130-091-221) was added to cells and then centrifuged again at 1000 rpm for 5 minutes to wash. Cells were resuspended in MACS buffer and then transferred to the flow cytometry tube. Labeling of each antibody was measured by a MACS Quant VYB (Miltenyi Biotec, 130-096-116) and graphs were produced with FlowJo software (FlowJo, LLC). This work was performed by the University of Michigan Cardiovascular Center Cardiovascular Regeneration Core Laboratory.

**Immunofluorescence staining**

Pluripotency immunofluorescence

DMD and control iPSCs were plated on sterile glass coverslips coated with 100 µg/mL Matrigel (Corning, 354277) in DMEM/F12 (Life Technologies,11330032) and cultured in StemMACS™ iPS-Brew XF (Miltenyi Biotec, 130-104-368) at 37°C and 5% CO2. When colonies formed, iPSCs were fixed with 4% paraformaldehyde in PBS at 4°C for 20 minutes. Following fixation, iPSCs were washed twice with PBS for 5 minutes and then blocked for 1 hour with block solution containing 1% BSA and 0.1% Triton X-100 in PBS. After blocking, iPSCs were labeled with primary antibodies recognizing pluripotency markers Nanog (R&D Systems, AF1997), Oct4 (Cell Signaling, 2750), and Sox2 (R&D Systems, AF2018) diluted 1:100. iPSCs were incubated overnight at 4°C with each primary antibody in block solution. The next day, all coverslips were washed three times with PBS for 5 minutes. Next, labeled iPSCs were incubated with secondary antibodies Cy3 AffiniPure Goat Anti-Rabbit IgG (Jackson ImmunoResearch, 111-165-144) or Cy3 AffiniPure Donkey Anti-Goat IgG (Jackson ImmunoResearch, 705-165-147) were diluted 1:400. DAPI (Sigma Aldrich, D9542) 1:10,000 was added to all secondary antibody dilutions. iPSCs were incubated for 1 hour at room temperature with each secondary antibody in block solution. Next, all coverslips were washed three times with PBS for 5 minutes. Coverslips of immunostained iPSCs were mounted on microscope slides using Permafluor (Thermo Scientific, TA030FM). After drying, cells were imaged using an Olympus BX51 fluorescent microscope (Olympus, Tokyo, Japan) 20X objective to observe each pluripotency marker and nuclei.

iPSC derived cardiomyocyte dystrophin and troponin I immunofluorescence

Differentiated iPSC cardiomyocytes were dissociated, plated, fixed and immunostained as described in Lian et al. Nature Protocols Vol 8 No 1 2013 p 172. Troponin I is stained using Anti-Troponin I 1:500 (Millipore, MAB1691) and Cy3 AffiniPure Fab Fragment goat anti-mouse IgG 1:400 (Jackson ImmunoResearch,115-167-003). C-terminus of Dystrophin is labeled with MANDRA1 clone antibody 1:100 (Sigma Aldrich, D8043) and Cy3 AffiniPure rat anti-mouse IgG 1:200 (Jackson ImmunoResearch, 415-165-166). DAPI 1:10,000 stain was added to secondary antibody dilutions (Sigma Aldrich, D9542). All images were captured with an Olympus BX51 fluorescence microscope using 100X objective and oil immersion.

**Protein analysis**

iPSC and iPSC derived cardiomyocyte protein lysate preparation, SDS PAGE, and Western blotting of dystrophin and troponin I

Patient derived iPSCs, derived cardiomyocytes from iPSC cell lines, and adult left ventricle (LV) tissues were lysed in TBS (150 mM sodium chloride and 50 mM Tris-HCl, pH 7.5) containing 1% Triton X-100, 1 mM EDTA, and the following protease inhibitors: 0.5 mg/ml Pepstatin A, 2 kallikrein inhibitor units/ml Aprotinin, 1 mg/ml Leupeptin, 0.4 mM PMSF, and 0.6 mM Benzamidine. Lysates were centrifuged at 14,000 x g for 2 minutes, the supernatants were collected, and protein concentrations were quantified using a DC Protein Assay (Bio-Rad, 500-0112). 200 µg of total protein in 1XLSB was loaded per lane of a 3-15% polyacrylamide SDS gel and run at 55V overnight. Protein was transferred to a PVDF membrane at 100V for 3 hours. Western blotting proceeded using 5% nonfat dry milk in TBS + 0.5% Tween-20 for blocking and antibody incubations. Membranes were washed three times for 5 minutes with TBS + 0.5% Tween-20 between primary and secondary antibody incubations, and after secondary antibody incubation. Dystrophin was blotted with anti-dystrophin polyclonal 1:1000 (Abcam, ab15277) and then peroxidase-conjugated AffiniPure goat anti-rabbit IgG 1:1000 (Jackson ImmunoResearch, 111-035-144). cTnI & ssTnI are blotted with Anti-Troponin I 1:2000 (Millipore, MAB1691) and then peroxidase-conjugated AffiniPure goat anti-mouse IgG 1:1000 (Jackson ImmunoResearch, 115-035-146). DAPI 1:10,000 stain was added to secondary antibody dilutions (Sigma Aldrich, D9542). Chemiluminescence via substrate (Thermo Scientific, 34087) was captured with an AlphaEaseFC camera.

**iPSC derived cardiomyocyte protein lysates with WGA purification, SDS PAGE, and Western blotting of dystrophin**

One hundred µL of iPSC-CMs lysates (2.5 µg/µL) and adult left ventricle (LV) tissue lysates prepared previously were incubated with 33 µL of packed agarose bound wheat germ agglutinin (WGA) mixing overnight at 4°C. The next day, agarose-WGA was centrifuged at 2000 x g for 5 minutes. The void was removed and agarose-WGA was washed two times for five minutes mixing with washing buffer composed of TBS (150 mM sodium chloride and 50 mM Tris-HCl, pH 7.5) containing 0.1% Triton X-100 and the following protease inhibitors: 0.5 mg/ml Pepstatin A, 2 kallikrein inhibitor units/ml Aprotinin, 1 mg/ml Leupeptin, 0.4 mM PMSF, and 0.6 mM Benzamidine. Washes were removed with centrifugation, and then agarose-WGA was resuspended in 50 µL of 1XLSB in washing buffer and then boiled for 5 minutes. The agarose-WGA was centrifuged at 2000 x g for 5 minutes. The supernatant containing protein eluted from the agarose-WGA is the purified sample and was loaded onto 3-15% polyacrylamide SDS gel then run at 55V overnight. Protein was transferred to a PVDF membrane at 100V for 3 hours. Western blotting proceeded using 5% nonfat dry milk in TBS + 0.5% Tween-20 for blocking and antibody incubations. Membranes were washed three times for 5 minutes with TBS + 0.5% Tween-20 between primary and secondary antibody incubations, and after secondary antibody incubation. Dystrophin was blotted with anti-dystrophin polyclonal (Abcam, ab15277) and then peroxidase-conjugated AffiniPure goat anti-rabbit IgG (Jackson ImmunoResearch, 111-035-144). Chemiluminescence via substrate (Thermo Scientific, 34087) was captured with an AlphaEaseFC camera. The de-identified control human heart tissue was obtained from the National Disease Research Interchange (Philadelphia, PA USA) and is considered not regulated the University of Michigan Institutional Review Board. The Becker Muscular Dystrophy heart tissue was obtained from a failing heart at the time of heart transplant with study approval by the University of Michigan Institutional Review Board and informed consent of the subject. Both hearts were perfused with cardioplegia solution and kept on ice until dissection. The tissue was dissected and immediately flash frozen in liquid nitrogen followed by storage at -80C.

Table 2: Protein concentration in lysate samples

| **Lysate Sample I.D.** | **Conc. of total protein in**  **100 µL of lysate incubated with agarose-WGA** |
| --- | --- |
| Control iPSC-CMs | 269.20 µg |
| Carrier iPSC-CMs | 207.7 µg |
| DMD iPSC-CMs | 280.45 µg |
| Normal Human LV | 607.1 µg |
| Becker Human LV | 808.1 µg |

**Action potentials and I_f_ and I _Ca,L_ recording and analysis**

For action potential recordings, spontaneously contracting areas of monolayer culture were mechanically/enzymatically (0.25% trypsin-EDTA, Biological Industries, Beit-Haemek, Israel) dissociated and dispersed. This dispersion resulted in single cell-to-small clusters, which were plated on Matrigel-coated glass coverslips (13 mm diameter) in 24-well plates supplemented with 10 μM blebbistatin. Coverslips were incubated at 37°C for 2-4 days, followed by a 5 day recovery period without blebbistatin before performing electrophysiological experiments [9–11]. In all experiments, the coverslips were perfused at 37°C with an external solution containing (in mmol/l): 140 NaCl, 5.4 KCl, 1.8 CaCl_2_, 1 MgCl_2_, 10 glucose and 10 HEPES titrated to pH 7.4 with NaOH (310 mOsm). The patch pipette solution contained (mmol/l): 120 KCl, 1 MgCl_2_, 3 Mg-ATP, 10 HEPES, and 10 EGTA titrated to pH 7.2 with KOH and adjusted at 290 mOsm with saccharose (all materials were purchased from Sigma-Aldrich). Action potentials were recorded from cardiomyocyte clusters. I_f_ was recorded from single cardiomyocytes (enzymatically dissociated) in the presence of 500 μM BaCl_2_ to block I_K1_. To record I_f_, the membrane was clamped at 15 sec intervals, from a holding potential of -40 mV to -120 mV in 10 mV steps for 2 sec pulse durations. To record L-Type Ca^2+^ current (I_Ca,L_), the membrane was clamped at 200 ms depolarizing steps, from a holding potential of -70 mV ranging from -40 to +40 mV after a 20 ms -40 mV pulse for inactivation of Na^+^ currents [12]. Peak I_Ca,L_ was calculated as the difference between the maximal inward current amplitude to zero current level [13]. Axopatch 200B, Digidata 1322 or 1440 and pClamp10 (Molecular Devices, Sunnyvale, CA, USA) were used for data amplification, acquisition and analysis. Signals were digitized at 4-10 kHz. Patch electrodes with resistances of 4-7 MΩ were pulled from borosilicate glass capillaries (Harvard Apparatus, Holliston, MA, USA). The recordings were analyzed for the detection of all peaks of the recorded signal from which action potential parameters and IBIs were calculated by using the dedicated MATLAB software [9]. For current recording, pipette capacitance compensation was adjusted, whole cell capacitance was measured and series resistance compensation was adjusted to 80%; finally, current density was calculated. To generate current activation curves, conductance (G) values were calculated using the following reversal potential I_f_: -40 mV, I_ca, L_: DMD male, female, control 44 mV, 43 mV, 48 mV, respectively; according to the following relation: I=G(V-V_reversal_) [14].

**Micro Electrode Array (MEA) recordings**

Extracellular electrograms were recorded for 1000-1800 seconds from spontaneously contracting iPSC-CMs clusters by using the MEA apparatus (Multi Channels Systems, Reutlingen, Germany). Recordings were performed at a sampling frequency of 1000 Hz, downsampled to 200 Hz, and analyzed to detect peaks of the signal from which Inter-Beat Intervals (IBIs) were calculated by using a MATLAB software (Mathworks, Natick, MA, USA) [9].

**Transmission electron microscopy (TEM) of iPSC-CMs**

Samples were fixed by immersion in buffered glutaraldehyde 2.5%; the samples were post-fixed for 1 hr in buffered 1% OsO4 with 1.5% K4Fe(CN)6 (potassium ferrocyanide-reduced osmium). Fixed iPSC-CM clusters were embedded in 1% agar, dehydrated in graded ethanol series and further processed for epoxy resin (Agar100) embedded at 60°C for 48 hrs. One micrometer-thick sections (semithin sections) were stained with 1% toluidine blue and examined by light microscopy. The ultrathin sections were cut with a diamond knife at 60 nm thicknesses using a Leica ultramicrotome and double stained with 1% uranyl acetate and Reynolds lead citrate. Ultrastructural examination was performed with a Morgagni 286 transmission electron microscope (FEI Company, Eindhoven, The Netherlands) at 80 kV. Digital electron micrographs were recorded with a MegaView III CCD and iTEM-SIS software (Olympus, Soft Imaging System GmbH, Münster, Germany) was used for morphometry. The sarcomeres (distance between two consecutive Z bands) were measured on 10 cells having at least two sarcomeres for each group.

**Statistical analysis**

Results are presented as mean±SEM. Comparisons between Male-DMD, Female-DMD and control iPSC-CMs were performed using One-Way or Two-Way ANOVA followed by Holm-Sidak test using SigmaPlot 12.0 software (Systat Software International, San Jose, CA, USA). A value of P<0.05 was considered statistically significant.

**References**

[1] **Takahashi K, Yamanaka S**. Induction of pluripotent stem cells from mouse embryonic and adult fibroblast cultures by defined factors. *Cell* 2006; 126; 663–76.

[2] **Novak A, Barad L, Lorber A, et al.** Functional abnormalities in iPSC-derived cardiomyocytes generated from CPVT1 and CPVT2 patients carrying ryanodine or calsequestrin mutations. *J. Cell. Mol. Med.* 2015; 19; 2006–18.

[3] **Novak A, Barad L, Zeevi-Levin N, et al.** Cardiomyocytes generated from CPVT D307H patients are arrhythmogenic in response to β-adrenergic stimulation. *J. Cell. Mol. Med.* 2012; 16; 468–82.

[4] **Yehezkel S, Rebibo-Sabbah A, Segev Y, et al.** Reprogramming of telomeric regions during the generation of human induced pluripotent stem cells and subsequent differentiation into fibroblast-like derivatives. *Epigenetics* 2011; 6; 63–75.

[5] **Ben Jehuda R, Eisen B, Shemer Y, et al.** CRISPR correction of the PRKAG2 gene mutation in the patient’s iPSC-derived cardiomyocytes eliminates the electrophysiological and structural abnormalities. *Hear. Rhythm* 2017; DOI: 10.1016/j.hrthm.2017.09.024.

[6] **Lian X, Zhang J, Azarin SM, et al.** Directed cardiomyocyte differentiation from human pluripotent stem cells by modulating Wnt/β-catenin signaling under fully defined conditions. *Nat. Protoc.* 2013; 8; 162–75.

[7] **Kim D, Pertea G, Trapnell C, et al.** TopHat2: accurate alignment of transcriptomes in the presence of insertions, deletions and gene fusions. *Genome Biol.* 2013; 14; R36.

[8] **Trapnell C, Williams BA, Pertea G, et al.** Transcript assembly and quantification by RNA-Seq reveals unannotated transcripts and isoform switching during cell differentiation. *Nat. Biotechnol.* 2010; 28; 511–5.

[9] **Ben-Ari M, Schick R, Barad L, et al.** From beat rate variability in induced pluripotent stem cell-derived pacemaker cells to heart rate variability in human subjects. *Heart Rhythm* 2014; 11; 1808–18.

[10] **Mandel Y, Weissman A, Schick R, et al.** Human embryonic and induced pluripotent stem cell-derived cardiomyocytes exhibit beat rate variability and power-law behavior. *Circulation* 2012; 125; 883–93.

[11] **Herron TJ, Da Rocha AM, Campbell KF, et al.** Extracellular matrix-mediated maturation of human pluripotent stem cell-derived cardiac monolayer structure and electrophysiological function. *Circ. Arrhythmia Electrophysiol.* 2016; 9.

[12] **Hwang HS, Kryshtal DO, Feaster TK, et al.** Comparable calcium handling of human iPSC-derived cardiomyocytes generated by multiple laboratories. *J. Mol. Cell. Cardiol.* 2015; 85; 79–88.

[13] **Tiaho BYF, Nargeot J, Richard S, et al.** Repriming of l-type calcium currents revealed during early whole-cell patch-clamp recordings in rat ventricular cells. *J. Physiol.* 1993; 367–89.

[14] **Ma J, Guo L, Fiene SJ, et al.** High purity human-induced pluripotent stem cell-derived cardiomyocytes: electrophysiological properties of action potentials and ionic currents. *Am. J. Physiol. Circ. Physiol.* 2011; 301; H2006–17.
